# Deadlines make you productive, but what do they do to your motivation? Trajectories in quantity and quality of motivation and study activities among university students as exams approach

**DOI:** 10.3389/fpsyg.2023.1224533

**Published:** 2023-12-05

**Authors:** Jan Dirk Capelle, Kerstin Senker, Stefan Fries, Axel Grund

**Affiliations:** ^1^Department of Psychology, Bielefeld University, Bielefeld, Germany; ^2^Institute of Psychology, University of Duisburg-Essen, Essen, Germany; ^3^Luxembourg Centre for Educational Testing (LUCET), University of Luxembourg, Esch-sur-Alzette, Luxembourg

**Keywords:** LGCM, parallel process model, motivation, expectancy, value, motivational conflict, deadline, temporal landmark

## Abstract

**Introduction:**

Recent research has emphasized that achievement motivation is context-sensitive and varies within individual students. Ubiquitous temporal landmarks such as exams or deadlines are evident contextual factors that could systematically explain variation in motivation. Indeed, research has consistently found that university students increase their study efforts as exams come closer in time, indicating increasing study motivation. However, changes in study motivation for a specific exam as it comes closer have rarely been investigated. Instead, research on developmental changes in expectancy and value beliefs has consistently founds that achievement motivation declines over a semester. Surprisingly, declining motivation thus apparently coincides with increasing study efforts for end-of-semester exams.

**Methods:**

The present research investigates this apparent contradiction by assessing how exam-specific motivation and study behavior change under equal methodological conditions as an exam draws closer. Using parallel growth curve models, we examine changes in expectancy-value beliefs, performance approach and avoidance motivation and study behavior as well as motivational want- and should-conflicts among 96 students over eight weekly measurement points.

**Results and discussion:**

Results show that students study more for their exam as it comes closer and increase their use of surface learning strategies more rapidly than their use of deep learning strategies. However, even exam-specific expectancy and attainment value beliefs decline while performance-avoidance motivation increases over time, indicating that students increasingly study out of fear to fail as exams come closer. Consistent with these findings, students’ experience of should conflicts decreases while their want conflicts increase over time. We discuss several possible mechanisms underlying our findings in addition to potential theoretical consequences and suggest future research opportunities to better understand students’ changes in situative motivation and study behavior in the context of temporal landmarks.

## Introduction

Temporal landmarks like assignment deadlines and exams are ubiquitous in university students’ lives and students consequently adjust their study activities according to these deadlines (Peetz and Wilson, 2013). Specifically, students often increase their study efforts as a temporal landmark draws closer and even” cram” or “rush” their study tasks right before their respective deadline (Schouwenburg and Groenewoud, 2001; König and Kleinmann, 2005; Blasiman et al., 2017; Liborius et al., 2019).

Recent research on achievement motivation has started to increasingly emphasize the importance of situational motivation for a specific study task (Hulleman et al., 2010; Richey et al., 2018; Eccles and Wigfield, 2020; Wigfield and Eccles, 2020). Despite the ubiquity and evident relevance of exams in students’ lives, very little is known about how students’ exam-specific study motivation changes as they draw closer in time. Clearly, one would expect increasing study activities for an exam to be accompanied by increasing study motivation related to that exam. Interestingly, available studies on changes of study motivation consistently found that expectancy (“can I do this task?”) and value (“why should I do this task?”) measures of motivation tend to decline over time (e.g., Sonnert et al., 2015; Kosovich et al., 2017; Robinson et al., 2019). At first sight, this is surprising, as higher study efforts should be expected to be accompanied by higher study motivation. One evident explanation for this apparent discrepancy are methodological differences. For example, temporal changes in achievement motivation are often assessed as more general expectancy and value beliefs toward a class or study course over longer time periods such as one or several semesters rather than study motivation for a specific study task related to an exam (e.g., Dresel and Grassinger, 2013; Kosovich et al., 2017; Dietrich et al., 2019). In contrast, study behavior before an exam is usually assessed over much shorter time periods such as days or weeks and with regard to a specific exam (e.g., Liborius et al., 2019; Capelle et al., 2022).

The present research bridges these distinct parts of the literature by investigating the intraindividual trajectories of students’ study motivation and study activities for a specific upcoming exam in a methodologically consistent way. We thereby attempt to answer two overarching questions: First, how do study motivation and study behavior change as an exam comes closer? Secondly, do changes in directly measured study motivation covary systematically and positively with changes in study behavior?

In order to gain a comprehensive picture of the temporal dynamics of study motivation and study activities and to illuminate the apparent contradiction between declining expectancy-value beliefs and increasing study efforts, we consider changes in a set of variables from different research traditions to explain students’ motivation and study behavior. First, in addition to expectancy and task value beliefs as a measure focusing on *how much* students are motivated (i.e., the quantity of their motivation), we also consider students’ performance-approach and performance-avoidance goals as measures focusing on *why* they are motivated to study (i.e., the quality of their motivation). As such, we consider study motivation in terms of two central dualities in motivation research: That of expectancy and value beliefs as well as that of approach and avoidance tendencies (e.g., Elliot, 1999; Hulleman et al., 2008). Similarly, in addition to assessing students’ total study time as measure focusing on *how much* they engage in studying (i.e., the quantity of their study activities), we consider their use of cognitive learning strategies as a measure focusing on *how* they study (i.e., the quality of their study activities). Finally, we consider motivational want- and should conflicts as measures of latent motivation combining both its quantitative and qualitative aspects (Riediger and Freund, 2008; Grund et al., 2015a; Capelle et al., 2022). Motivational conflicts occur when students feel like they would prefer to or feel like they ought to perform a different activity than the one they are currently pursuing. As such, motivational conflicts can be seen as an indicator for how students’ activities and their needs and priorities are aligned (Hofer and Fries, 2016). By jointly considering the trajectories of these variables over the same time period, we attempt to gain more nuanced insights into how changes in students’ study motivation and their study activities relate to each other as exams draw closer and to shed light on the apparent discrepancy in the declining trajectories of achievement motivation and increasing study activities above and beyond methodological differences. To the best of our knowledge, this study is the first to consider changes of study behavior and study motivation relating to a temporal landmark (i.e., an exam) simultaneously and in a longitudinal study design over actual passing time.

### (Situative) expectancy and value beliefs as measures of motivation

Within educational psychology, expectancy and value beliefs are widely distinguished to describe two fundamental dimensions of students’ motivation and comprehensively explain achievement-related behavior (Wigfield and Eccles, 2000; Kosovich et al., 2015; Hulleman et al., 2016). Expectancy describes the subjective overall confidence to perform a task successfully (“can I do this task?”) while perceived overall task value describes the subjective overall desirability to perform a study task (“why should I engage in this task?”; e.g., Atkinson, 1957; Eccles, 1983; Duncan and McKeachie, 2005; Nagengast et al., 2011; for overviews, see Wigfield and Cambria, 2010; Barron and Hulleman, 2015; Hulleman et al., 2016; Eccles and Wigfield, 2020). Expectancy and task value beliefs provide a comprehensive framework to describe the overall degree to which an individual is motivated to pursue an activity such as a study task and thus energize study behavior (Atkinson, 1964; Eccles and Wigfield, 2002; Wigfield and Cambria, 2010; Kosovich et al., 2015; Hulleman et al., 2016). In tandem, expectancy and value can thus be seen as indicating *how much* an individual is motivated to pursue a task, which we call abbreviately “motivation quantity” in the present research.

Within contemporary expectancy-value research, there are two distinctions of importance to the current research. First, task value is routinely distinguished into the three positively valenced subfacets intrinsic value (“how enjoyable or interesting is the task?”), attainment or importance value (“how important is the task?”), utility value (“how useful is the task for my future goals?), and the negatively valenced subfacet cost (“how much effort requires a task?” or “how much time or resources does the activity take away from other tasks?”; Eccles et al., 1983; Eccles and Wigfield, 2020). While recent research has started to disentangle the differential contributions of these subfacets to various academic outcomes (e.g., Perez et al., 2019; Benden and Lauermann, 2022; Beymer and Rosenzweig, 2023), the positive-valenced subfacets of task value are often highly correlated and thus routinely summarized into one task value score (e.g., Dresel and Grassinger, 2013; Johnson et al., 2014; Perez et al., 2019 for discussions and overviews, see Kosovich et al., 2015; Hulleman et al., 2016; Eccles and Wigfield, 2020; Wigfield and Eccles, 2020). In the present research, we focus on changes in attainment or importance value rather than the full spectrum of value subfacets. This focus seemed prudent in order to reduce the burden on participants and the risk of systematic study dropouts (e.g., Loo, 2002; Bergkvist and Rossiter, 2007) and is fairly common in longitudinal research designs (e.g., Sonnert et al., 2015; Kosovich et al., 2017). Moreover, subjective importance of studying for the exam seemed to capture the notion of “motivation quantity” well and to be a likely and interesting subfacet to change in relation to a temporal landmark (see below for a further discussion).

The second distinction pertains to the level or specificity at which motivational beliefs are measured: While expectancy and value beliefs conceptually relate to a specific activity or task (Eccles et al., 1983; Wigfield and Cambria, 2010; Eccles and Wigfield, 2020; Wigfield and Eccles, 2020; *cf.* Eccles and Wigfield, 2002), they are often assessed in more general terms as achievement motivation relating either to a particular class or to a study domain rather than a specific task (Dietrich et al., 2019; Parrisius et al., 2022).The relationship between these more general motivational beliefs and academic outcomes is well established. Higher levels of expectancy and value beliefs regarding a study domain or a course of study (e.g., math or chemistry) are predictive of achievement outcomes such as the choice of a course of study (Battle and Wigfield, 2003; Eccles, 2005; Musu-Gillette et al., 2015), performance (Trautwein et al., 2012; Perez et al., 2019; for an overview, see Wigfield and Eccles, 2020), as well as drop-out intentions (Schnettler et al., 2020). Indeed, constructs that are conceptually and empirically similar to expectancy such as self-efficacy and academic self-concept are among the strongest predictors of academic performance (Eccles and Wigfield, 2002; for meta-analyses, see Robbins et al., 2004; Richardson et al., 2012). Similarly, higher levels of expectancy-value beliefs regarding a particular class as well as their changes over time are associated with performance and interest in that class (Tanaka and Murayama, 2014; Kosovich et al., 2017; Perez et al., 2019; Benden and Lauermann, 2022).

Recent research has increasingly emphasized the measurement of more situation- or task-specific study motivation and its relationship with academic outcomes (e.g., Dietrich et al., 2017; Berweger et al., 2022). Underlying this trend is the recognition that a students’ motivation regarding specific tasks or contents can differ between tasks and situations (Tsai et al., 2008; Tanaka and Murayama, 2014; Dietrich et al., 2019; Parrisius et al., 2022; Beymer and Rosenzweig, 2023). Indeed, Eccles and Wigfield recently re-named their expectancy-value theory into situative expectancy value theory (SEVT) in order to emphasize the conceptual focus on the situational genesis of study motivation (Eccles and Wigfield, 2020). This emphasis of situative motivation has sparked a greater interest in the contextual factors that contribute to different motivational states within a person at different points in time (e.g., Benden and Lauermann, 2022; Parrisius et al., 2022; Beymer and Rosenzweig, 2023). Currently available research suggests that higher levels of task- or content-specific motivational beliefs are positively associated with outcomes such as study effort (Dietrich et al., 2017) and positive emotions (Berweger et al., 2022). However, we are not aware of research investigating whether changes in motivational beliefs regarding a specific task covary with actual study activities relating to that task, although such a covariation seems central to expectancy and task value (e.g., Wigfield and Cambria, 2010; Hulleman et al., 2016). Conceptually, one should expect that systematic changes in expectancy and value over time covary systematically and positively with changes in study behavior: If students’ expectancy and value beliefs increase, so should their study efforts, and vice versa (Dietrich et al., 2017; Eccles and Wigfield, 2020; Capelle et al., 2022; Parrisius et al., 2022). Moreover, to our knowledge, temporal landmarks such as deadlines or exams have rarely been considered as situational factors that might explain systematic changes in situative motivation and study behavior, although there are both theoretical and empirical reasons to assume that they are highly relevant for students’ study activities, and by extension, their study motivation. In the present research, we consequently address the relationship of situative expectancy-value beliefs regarding a specific study task (i.e., studying for an exam) with their respective study activities and the temporal proximity of an exam as a potential situational factor that might help to explain their systematic (co-)variation. This approach to assessing systematic changes in study motivation is novel in educational psychology and can only draw on very limited conceptual or empirical work. Specifically, we are not aware of theoretical models considering the effect of approaching temporal landmarks. We therefore draw on temporal discounting models from other fields in psychology and economics to theoretically inform our hypotheses (Grüne-Yanoff, 2015).

### Motivation and temporal landmarks

Theoretical models concerned with systematic changes of motivation with regard to a temporal landmark converge on the prediction that study motivation and study activities increase as temporal landmarks such as exams or deadlines draw closer in time (Liberman and Förster, 2008; Capelle et al., 2022). The assumed psychological mechanism underlying these models is temporal discounting (Ainslie, 1992; Frederick et al., 2002).

Temporal discounting refers to a decreasing subjective valuation of outcomes the farther they are away in time and has been demonstrated in a myriad of paradigms (for overviews, see Frederick et al., 2002; Trope and Liberman, 2003; for recent reviews, see Odum et al., 2020; Seaman et al., 2022). Conversely, events or outcomes that are closer in time are subjectively higher valued. For example, passing an exam might become subjectively more valuable if it is only one week away compared to four weeks, thus increasing the subjective valuation of study activities (e.g., Steel and König, 2006; Urminsky and Goswami, 2015; Olsen et al., 2018). In the case of the present research, temporal discounting thus means that the subjective importance (i.e., attainment value) of studying for an upcoming exam should increase as the exam comes closer.

Temporal Motivation Theory, which is explicitly based on both expectancy value theories and temporal discounting, expands this perspective by incorporating expectancy beliefs and predicts that study motivation for an upcoming exam increases as it comes closer (Steel and König, 2006; Steel, 2007). Specifically, “motivation can be understood by the effects of expectancy and value, weakened by delay […]” (Steel and König, 2006, p. 897), where delay denotes the distance of a relevant temporal landmark. Consequently, study motivation as well as actual study activities should increase with an approaching deadline or exam. Due to its explicit reference to expectancy and value beliefs, Temporal Motivation Theory can be readily related to changes in expectancy and value measures of motivation over time: Both expectancy and value beliefs regarding studying for an exam should increase (Steel and König, 2006, but see Steel and Weinhardt, 2018 for a differentiation).

#### Expectancy and value over time

As previously mentioned, temporal changes in expectancy-value beliefs regarding a class or a course of study have been empirically assessed in terms of developmental changes (e.g., Wigfield, 1994) or after performance feedback (e.g., Benden and Lauermann, 2022) rather than changes of study motivation for a specific study task related to the proximity of a temporal landmark. Still, considering this research is likely informative for two reasons: First, levels of more general expectancy-value beliefs regarding a class or a study domain are likely correlated with expectancy-value beliefs for specific study tasks (Hulleman et al., 2016; Dietrich et al., 2019). For example, it seems more likely that a student with high motivation for a class or their studies in general at the end of the semester reports high study motivation for a specific study task at that time than a student with low motivation for a class. Indeed, it seems plausible that students’ task-specific motivation various around their class- or domain-specific motivation. Secondly, many longitudinal studies assessed changes in expectancy and value over one full semester (e.g., Dresel and Grassinger, 2013; Perez et al., 2019). Hence, the end of the assessment period often coincides with relevant “high stakes” deadlines at the end of a semester, although such deadlines can already appear during a semester (e.g., Muis and Edwards, 2009; Benden and Lauermann, 2022). It thus seems plausible that motivational effects of exams are to some degree reflected in these studies.

However, studies that directly assessed the development of students’ achievement motivation over one semester almost unanimously found a significant decline in both expectancy and value beliefs over time, regardless of differences in measurement specificity and operationalization (Zusho et al., 2003; Dresel and Grassinger, 2013; Johnson et al., 2014; Sonnert et al., 2015; Kosovich et al., 2017; Kaplan et al., 2018; Robinson et al., 2019; Benden and Lauermann, 2022, with the exception of high performing students; for descriptive declines, see Perez et al., 2019; see Grays, 2013, for statistically non-significant changes). Decreases in expectancy and value beliefs over time are also well-documented among school children (for a recent meta-analysis, see Scherrer and Preckel, 2019). Even considering the methodological caveats mentioned above, these findings seem to be contrary to the predictions of the temporal discounting models, warranting further investigation.

#### Study quantity over time

The decline in class- or domain-specific expectancy and value beliefs over time becomes even somewhat more surprising when considering the changes in the time students spend studying (i.e., the quantity of study activities) over time: In concordance with theoretical predictions, research that directly assessed students’ study time in relation to a temporal landmark consistently found that students’ study activities increase toward exam dates at the end of the semester. These increases are sometimes dramatic and referred to as “cramming” (Schouwenburg and Groenewoud, 2001) or “deadline rush” (Steel and Weinhardt, 2018). Indeed, Taraban et al. (1999) investigated the pattern of study behavior based on self-report measures and data trails for three weeks and reported that students studied mainly just before exams (see also Blasiman et al., 2017). The increase in study efforts toward a temporal landmark is fairly universal, even if the exact shape of the trajectory of study quantity varies somewhat in the literature, presumably depending on the time period covered (Schouwenburg and Groenewoud, 2001). For example, Liborius et al. (2019) reported that students’ learning time trajectories over one full semester were best described by a quadratic function. In a reanalysis of the data used by Taraban et al. (1999) and König and Kleinmann (2005) found that the increase in study activities followed a hyperbolic increase in a different study over ten weeks, which was also reported by Dewitte and Schouwenburg (2002). Similarly, Capelle et al. (2022) reported that university students’ probability to engage in study activities increased exponentially over two weeks before exams. This unambigious constellation of findings suggests that temporal landmarks can be considered “strong” situations in the sense that they elicit fairly similar behavior (i.e., increasing their study quantity) among most individuals (Mischel, 1977; Fleeson, 2007; Peetz and Wilson, 2013; Dweck, 2017). In the present research, we therefore expected students’ study quantity to increase as an exam comes closer in time.

### Can quality help explain differences in quantity?

In summary, the available evidence suggests that students’ study or work time (i.e., study quantity) increases substantially as a temporal landmark such as a deadline or exam comes closer, while general measures of expectancy and value beliefs (i.e., motivation quantity) generally decrease over time. Study behavior thus seems to match theoretical predictions by temporal discounting and Temporal Motivation Theory while currently available evidence regarding expectancy-value beliefs do not. The above-mentioned differences in how expectancy and value are measured (i.e., in more general terms rather than with regard to specific study tasks related to a temporal landmark) offers one plausible explanation for this constellation of findings, which we address in the present research. Additionally, considering changes in constructs beyond expectancy-value measures of motivation and absolute study time are likely to be useful in gaining a more comprehensive picture of changes in motivation and study activities as a temporal landmark comes closer:

Regarding motivation, Temporal Motivation Theory – while primarily focused on expectancy and value beliefs (Steel, 2007) – explicitly recognizes that approach and avoidance incentives (i.e., the motivation quality) are likely processed differently over time (Steel and König, 2006; Steel and Weinhardt, 2018). As such, it incorporates ideas from older theories concerned with intra-personal conflicts between approach and avoidance tendencies (e.g., Lewin, 1938; Dollard and Miller, 1950; Epstein, 1978). Moreover, regarding study activities, it is important to note that simply investing more study time is not necessarily associated with better grades (Purdie and Hattie, 1999; Enders and Weinzierl, 2017). Instead, *how* a student studies (i.e., the quality of their study activities) is likely relevant (Duncan and McKeachie, 2005; Hattie and Donoghue, 2016). In order to gain a more thorough understanding of the temporal dynamics of students’ motivation, it thus makes sense to also consider the trajectories of qualitative aspects of motivation and study activities as a temporal landmark comes closer. We now introduce our conception of motivation quality and available evidence regarding its change over time, followed by study quality.

#### Quality of motivation: performance approach vs. avoidance

Most motivation theories distinguish in some way between the quantity of motivation (i.e., *how much* a student is motivated overall) and the quality of motivation (i.e., *why* a student is motivated to pursue a specific study activity; e.g., Grund, 2013; Hofer and Fries, 2016; Senko, 2016). These qualitative differences pertain to different incentives (i.e., the value component as opposed to the expectancy component in an expectancy-value framework; Bong, 2001; Grund, 2013; Hulleman et al., 2016). For the purpose of this study, we consider qualitative differences of motivation in terms of the fundamental duality of approach and avoidance motivation (Elliot and Thrash, 2001; Harackiewicz et al., 2002; Elliot, 2006; Elliot and Friedman, 2007; Feltman and Elliot, 2012). Specifically, we consider the distinction between performance approach and performance avoidance goals (Elliot and Harackiewicz, 1996; Elliot, 1999): If a student studies because they hope to gain something such as demonstrating their knowledge by outperforming others, they are motivated by a performance approach goal. If a student studies because they want to avoid negative consequences such as failing an exam, they are motivated by a performance avoidance goal (Elliot, 2006; Elliot and Friedman, 2007; Bjørnebekk and Gjesme, 2009; Feltman and Elliot, 2012).^1^ We considered a focus on performance approach and performance avoidance goals appropriate, as they are valid and widely used constructs to assess the approach-avoidance duality in educational psychology (e.g., Elliot and Church, 1997; Cury et al., 2006; Senko et al., 2011; Scott et al., 2015) and seem particularly well-suited in the context of an upcoming exam as a “high-stakes” performance test (Harackiewicz et al., 2002; Cole and Osterlind, 2008; Darnon et al., 2009).

Previous research has consistently linked more general (i.e., trait-like regarding an overall study course or domain; Barron and Harackiewicz, 2003) performance-avoidance goals with worse performance- and wellbeing-outcomes (Murayama et al., 2011; Senko et al., 2011; for overviews, see DeShon and Gillespie, 2005; Wigfield and Cambria, 2010). In contrast, performance approach goals are mostly associated with better performance-outcomes and, in some cases, wellbeing-outcomes (Harackiewicz et al., 1998; Senko et al., 2011; Urdan and Kaplan, 2020; for meta-analyses, see Hulleman et al., 2010; Huang, 2012), but not uniformly so (e.g., Midgley et al., 2001; Senko, 2016; Miller et al., 2021), likely due to differences in operationalizations (Hulleman et al., 2010; Senko et al., 2011; Senko and Dawson, 2017).

Available research linking class-specific goals and performance such as final grades among college students has found that performance approach goals are positively related to class-performance, while performance avoidance goals are negatively liked to class performance (Elliot and Church, 1997; Church et al., 2001; Elliot and McGregor, 2001; Baranik et al., 2010). The same pattern can be found regarding associations between task-specific goals and task-performance (Elliot, 1999; Zusho et al., 2005; Pekrun et al., 2009; Baranik et al., 2010). Indeed, a meta-analysis of experimentally induced goals found that approach goals (including performance approach goals) were causally linked with better performance in laboratory settings (Van Yperen et al., 2015). Moreover, task-specific performance approach goals have been positively linked to positive achievement emotions and task interest, while task-avoidance goals are positively linked to negative achievement emotions and negatively linked to task interest (Zusho et al., 2005; Pekrun et al., 2009). By and large, the associations of performance approach and avoidance goals and relevant educational outcomes are consistent, regardless of level of specificity (Huang, 2012; Linnenbrink-Garcia et al., 2012; *cf.* Richey et al., 2018).

By considering performance approach and avoidance goals in addition to expectancy and attainment value, we focus on combining perspectives on motivation rather than considering other commonly used subfacets of task value (i.e., intrinsic and utility value as well as costs, e.g., Barron and Hulleman, 2015; Eccles and Wigfield, 2020), although these subfacets also consider different reasons why a student might study. We had several reasons for this decision: First and most fundamentally, considering the duality of approach and avoidance goals promises to be a conceptually meaningful addition to the duality of expectancy and value dimensions of motivation (Elliot and Covington, 2001; Elliot, 2006, 2023; Liem et al., 2008; Hulleman et al., 2016). Secondly, the positively-valenced subfacets of value are often highly correlated and generally positively related to educational outcomes, as outlined above (e.g., Eccles and Wigfield, 1995; Jacobs et al., 2002; Kosovich et al., 2015). As such, we considered it most promising to focus on expectancy and attainment value (i.e., subjective importance) as apparently valid measures of motivation quantity. By contrast, performance approach and avoidance goals are generally related to distinct educational outcomes, suggesting them to be a more meaningful measure of motivation quality (Harackiewicz et al., 2002; Murayama et al., 2011). Finally, the approach-avoidance distinction allowed us to make differential theoretical predictions on how these are discounted over time by drawing on older conflict theories.

#### Changes in performance approach and performance avoidance motivation over time

As previously mentioned, Temporal Motivation Theory recognizes different effects of approach and avoidance incentives without making specific predictions regarding their trajectories (Steel and König, 2006; Steel and Weinhardt, 2018). However, older conflict theories (Miller, 1944; Lewin, 1946; Losco and Epstein, 1974; Epstein, 1978; Shelley, 1994; for overviews, see Trope and Liberman, 2003; Steel and König, 2006) predict that approach and avoidance incentives develop differently over time: “The strength of avoidance increases more rapidly with nearness than does that of approach.” (Dollard and Miller, 1950, p. 352). Put differently, the discounting function of avoidance incentives is assumed to be steeper than that of approach incentives (Trope and Liberman, 2003). Whenever a temporal landmark is ambivalent (i.e., characterized both by approach and avoidance incentives), it is thus predicted that approach incentives are adopted to a relatively higher degree when the landmark is further away and avoidance incentives are increasingly adopted to a relatively higher degree as it comes closer in time (Trope and Liberman, 2003). In the present case of an exam and performance goals, this would mean that the goal to perform better than others is more likely to be salient when the exam is farther away and the goal to avoid failure becomes more salient as the exam draws closer.

Direct empirical evidence for differential discounting functions of approach and avoidance incentives relies on operationalizing them in terms of gains and losses of monetary rewards, respectively. While Mogilner et al. (2007) report evidence that avoidance goals are indeed discounted more steeply, other studies found evidence to the contrary, i.e., that losses are discounted *less* steeply than gains (Loewenstein, 1987; Benzion et al., 1989; for a review, see Frederick et al., 2002).

Studies that investigated changes in university students’ performance approach and avoidance goals longitudinally over actual passing time are rare and show similarly contradictory results: Task-specific performance approach and avoidance goals were found to increase, decrease, and remain stable between different tasks both when they were task-specific (e.g., exams or writing assignments; Fryer and Elliot, 2007; Muis and Edwards, 2009; Han, 2016) and course-specific (Senko and Harackiewicz, 2005; Jagacinski et al., 2010). Overall, however, the adoption of performance avoidance goals appears to be relatively more likely to increase over time and the adoption of performance approach goals more likely to be either stable or decline (Senko and Harackiewicz, 2005; Fryer and Elliot, 2007; Muis and Edwards, 2009). Similar patterns have been found for trait-like goal orientations (Dresel and Grassinger, 2013; for an overview among school children, see Scherrer and Preckel, 2019). The available evidence thus suggests a pattern of changes that is broadly compatible with the predictions of conflict theories. However, changes in goal adoption were mostly considered *between* different exams or assignments rather than with reference to an upcoming temporal landmark (e.g., Senko and Harackiewicz, 2005; Fryer and Elliot, 2007; Muis and Edwards, 2009). As such, these relative changes in goal adoption that were found likely reflect effects of direct performance feedback rather than effects of temporal proximity (Senko and Harackiewicz, 2005; Benden and Lauermann, 2022) and can thus inform the present research only to a limited degree.

As the present research is, to the best of our knowledge, the first to address potential changes among performance approach and avoidance goals as a relevant exam comes closer, and against the backdrop of the inconclusive state of the empirical evidence just outlined, we base our predictions on changes in motivation quality mainly on the theoretical arguments made by conflict theories (e.g., Trope and Liberman, 2003). We now consider the quality of study activities.

#### Quality of study activities: surface vs. deep learning strategies

Study time can be spent pursuing qualitatively different activities, which are commonly categorized into learning strategies (e.g., Pintrich et al., 1993). Learning strategies thus describe *how* students study. Different types of learning strategies have been proposed and investigated in the context of self-regulated learning (e.g., Pintrich et al., 1993; Pintrich, 2004). We focus on cognitive learning strategies because they are concerned with the actual processing of learning material. Moreover, the distinction between surface and deep learning strategies (also referred to as elaboration strategies) captures the idea of different qualities of study activities particularly well (e.g., Gow and Kember, 1990; Biggs et al., 2001; Duncan and McKeachie, 2005; Justicia et al., 2008). Surface strategies refer to learning activities such as memorizing disjointed pieces of information over a short time period, typically with the goal of reproducing them in order to pass an exam (Gow and Kember, 1990; Entwistle, 1998). In contrast, deep learning strategies refer to learning activities such as coming up with new examples and finding connections between different concepts, typically with the goal of gaining a deep and long-term understanding of the respective study content (e.g., Vermunt, 1998; Zlatović et al., 2015). While the use of deep learning strategies instead of surface learning strategies is sometimes associated with better learning outcomes (e.g., Duncan and McKeachie, 2005; *cf.* Zusho et al., 2003), the adaptive choice of learning strategies suitable for a specific task is likely more relevant for learning outcomes than the use of any single strategy (Purdie and Hattie, 1999; Credé and Phillips, 2011; Hattie and Donoghue, 2016; Stark, 2019). Moreover, both the quantity (i.e., expectancy and value; Pintrich et al., 1993; Berger and Karabenick, 2011; Credé and Phillips, 2011) and the quality of students’ motivation (i.e., the degree to which students adopt performance approach or avoidance goals) are related to their use of different learning strategies (Elliot, 1999; Elliot and McGregor, 2001; Liem et al., 2008; for a review, see Senko et al., 2011).

#### Surface and deep learning strategies over time

There is, again, only little empirical research on how students’ use of learning strategies changes over actual passing time that has so far produced ambiguous findings. Studies by Enders and Weinzierl (2017) over one semester and Naujoks and Händel (2020) over nine weeks found that students tend to use less deep learning strategies and more surface strategies as an exam comes closer in time. In contrast, Zusho et al. (2003) found that cognitive surface learning strategies decreased over 15 weeks, while deep learning strategies both increased (organization) and decreased (elaboration) and that trajectories varied with students’ achievement level.

Again, the current state of research warrants no clear expectations regarding changes in the use of surface and deep learning strategies as a temporal landmark draws closer. Theoretically, if one assumes that study quantity increases, as we expected in the current study, one might also expect the usage of both surface and deep learning strategies to increase (Busato et al., 1998; Vermunt, 1998; Berger and Karabenick, 2011). On the other hand, performance-avoidance goal adoption, which we assume to increase as an exam comes closer, is associated with the usage of surface learning strategies (Elliot, 1999; Senko et al., 2011). Accordingly, one might expect only surface learning strategies to increase as an exam draws closer. Given that the available empirical evidence both supports and contradicts such theoretical considerations, we assumed that students’ usage of learning strategies likely changes as an exam comes closer but had no clear expectation regarding the direction of change.

#### Multiple action alternatives and relative study motivation: motivational conflicts

Our final theoretical approach to investigate motivational changes over time is the perspective of multiple goals. One assumption in much of the research on study motivation and learning strategies is that students are not necessarily motivated for only one thing at a time (e.g., Atkinson and Birch, 1970; Boekaerts and Corno, 2005; Eccles, 2009). Importantly, this assumption implies that high study motivation regarding a study activity by itself (“absolute study motivation”) is a necessary but not sufficient condition to actually engage in studying. Rather, study motivation has to be higher or stronger than the motivation to pursue other activities: A student might refrain from studying not because they lack study motivation but because they are more motivated to pursue a different activity (e.g., Atkinson and Birch, 1970; Eccles, 2009; Dweck, 2017; Eccles and Wigfield, 2020). The Theory of Motivational Action Conflicts (TMAC; Schmid et al., 2007; Fries et al., 2008; Hofer and Fries, 2016) focuses on situations in which students are similarly motivated for two or more mutually exclusive activities at once. According to the TMAC, such situations are characterized by motivational conflicts that can persist even when a decision for one of these activities has been made (Grund et al., 2015a; Brassler et al., 2016). Within an expectancy-value framework, an omitted activity (i.e., the activity that is not pursued) can be considered an opportunity cost (Eccles, 2005; Barron and Hulleman, 2015; Hofer and Fries, 2016). The experience of motivational conflicts can thus be seen as an indicator of the combined latent motivational tendencies for an activity relative to its alternatives (Grund and Fries, 2012; Grund, 2013). For example, motivational conflicts become more likely if a student is similarly motivated for different activities at once, whereas they are less likely if they are distinctly more motivated for one activity (Capelle et al., 2022). Indeed, the TMAC explicitly assumes that the configuration of both expectancy and value as well as approach and avoidance incentives for each activity influence the experience of motivational conflicts in a particular moment (Hofer and Fries, 2016). Based on these configurations, motivational conflicts can be distinguished into want and should conflicts. Experiencing a want-conflict is characterized as the desire to pursue a more enjoyable activity (e.g., “I would prefer to read an interesting book”), thus indicating that the competing (i.e., not pursued) activity is perceived more positively than the focal activity one is actually pursuing (e.g., Grund et al., 2015b). In contrast, a should-conflict is characterized by the belief that one should do something else (“I should tend to my studies”), thus indicating that the competing activity is perceived as more negatively (Grund et al., 2015b). The experience of either want- or should-conflicts can thus be seen as an indicator of both the latent motivational strength as well as valence of students’ motivational tendencies for the activities they pursue relative to their omitted (i.e., available but not pursued) action tendencies. As such, motivational conflicts provide additional insights into students’ latent motivation and their changes beyond directly measured motivational constructs pertaining to a specific (study) activity.

#### Motivational conflicts over time

The trajectories of motivational conflicts over time have rarely been investigated so far. Taking them into account could, however, be a valuable addition to the absolute measures of study motivation traditionally considered: If motivational conflicts are an indicator for the relative magnitude of motivational tendencies and if an exam that comes closer in time affects one of these tendencies (i.e., study motivation) systematically, this should be seen in changes in the nature, frequency, and intensity of students’ motivational conflict experiences. The frequency of motivational conflicts has indeed been shown to decrease as an exam comes closer in time, while the intensity of the motivational conflicts that still occurred increased (Capelle et al., 2022). The authors interpret these findings as indicators that students’ underlying study motivation increases relative to students’ motivation for other activities. As the study does not distinguish between want- and should-conflicts, these findings do not allow conclusions regarding the valence of students’ relative motivation. Still, the trajectories of overall motivational conflicts they found are compatible with theoretical predictions suggested by temporal discounting and the empirical findings on increasing study quantity over time (Ainslie, 1992; Hofer and Fries, 2016). In order to extend these results and to utilize motivational conflicts as indicators of possible changes in latent motivation, we consider changes in the experience of both want- and should conflicts in the present research.

## The present study

The objective of the current study is to provide a first investigation of two central questions. The first question is “how do motivation and study behavior systematically change as a temporal landmark (i.e., an upcoming exam) comes closer?.” Situative motivation (both in terms of expectancy-value beliefs and achievement goals) has been shown to vary considerably over time (Fryer and Elliot, 2007; Dresel and Grassinger, 2013; Hulleman et al., 2016; Dietrich et al., 2017; Kosovich et al., 2017; Parrisius et al., 2022). However, temporal landmarks such as deadlines or exams are rarely considered in the analyses, although their temporal approach likely explains some of these variations in situative motivation as well as study behavior (e.g., Capelle et al., 2022). Moreover, temporal discounting and in particular Temporal Motivation Theory and conflict theories offer a psychological mechanism that allows theoretical predictions regarding systematic changes in motivation and study behavior.

The second question is “do task-specific study motivation and task-specific study activities positively covary as an exam comes closer?”. Recall that the trajectories of study quantity but not those of motivation quantity match the theoretical predictions made by temporal discounting and Temporal Motivation Theory. However, as motivation as a construct is fundamentally concerned with explaining task-specific behavior such as achievement-related choices, intensity and persistence in studying, changes in (task-specific) motivation and behavior should be expected to positively covary (Eccles and Wigfield, 2020). As such, the present study investigates how well situative motivation “matches” study behavior before an exam.

We pursue several paths to answer these questions. First, there are several methodological differences in the current literature that could help explain the contradictory findings described above: Changes in constructs are often assessed over different periods of time. Recall that while changes in achievement motivation is often measured over one or more semesters, the quantity of study activities is often measured over much shorter time periods like days or weeks. Moreover, expectancy-value beliefs in particular are often operationalized as being domain- or course-specific rather than task-specific. While these levels of specificity and their changes are likely related, it seems plausible that their temporal dynamics differ: For example, it is conceivable that while a students’ class-specific motivation declines over time, their motivation to study for the upcoming exam might still increase as the exam comes closer. The current research takes these methodological aspects into account and thus models the trajectories of motivation and study activities “on equal terms.” Specifically, we assess study motivation in relation to a specific study-task related to a temporal landmark (i.e., an exam).

Secondly, we consider changes in performance approach and performance avoidance goals (i.e., motivation quality). Crucially, the approach-avoidance distinction is recognized by Temporal Motivation Theory (Steel and Weinhardt, 2018) and their differential changes as a temporal landmark comes closer is laid out in older conflict theories (Miller, 1944; Lewin, 1946; Epstein, 1978; Shelley, 1994). While previous empirical evidence regarding changes in performance goals of over time has found patterns that are broadly in line with these predictions, methodological differences limit the transferability of these findings to the present context. Thirdly, we consider changes in deep and surface learning strategies (i.e., study quality) over time in addition to study time (i.e., study quantity). Empirical evidence regarding their trajectories over time is mixed. Still, distinguishing how students study promises to further illuminate the dynamics of students’ motivation and studying before an exam – indeed, previous research has found that both motivation quantity and quality are related to study quality (e.g., Duncan and McKeachie, 2005). Fourth, we consider the trajectories of both motivational want- and should-conflicts as indicators for latent relative motivation over time, as outlined above. Given the novelty of the present approach and the sparsity of relevant literature, we formulate only two directed and three undirected hypotheses.

We found it difficult to formulate specific expectations regarding changes in expectancy-value beliefs. One the one hand, recall that while previous findings indicate declines in expectancy-value beliefs over time, they largely describe developmental changes of domain- or course-specific beliefs without explicit reference to a temporal landmark (e.g., Kosovich et al., 2017; Benden and Lauermann, 2022). On the other hand, a decline of expectancy-value beliefs regarding task-specific study activities connected to a temporal landmark (as is the case in the present research) would contradict both theoretical predictions and the empirical findings regarding increasing study quantity over time, assuming that higher exam-related expectancy and value beliefs predict higher exam-related study effort. Indeed, one of the very reasons for our investigation was to find out if controlling for these methodological differences might “reconcile” changes in motivation and study behavior, as we outlined above. We had no clear foundation to decide which of these arguments should be given greater consideration in order to warrant a directed hypothesis. Consequently, we tested changes in expectancy-value beliefs as measures of motivation quantity without an assumed direction:

*H1*: Students’ motivation quantity regarding an exam changes as the exam comes closer in time.

*H1a*: Students’ expectancy regarding an exam changes as the exam comes closer in time.

*H1b*: Students’ attainment value regarding an exam changes as the exam comes closer in time.

The case seemed a bit clearer with regard to motivation quality: Here, the evidence and theoretical considerations seemed more in line, as we have illustrated above. We thus expected the overall quality of study motivation to decline. Specifically, we expected performance approach motivation to decline and performance avoidance motivation to increase over time:

*H2*: Students’ quality of study motivation regarding an exam declines as the exam comes closer in time.

*H2a*: Students’ performance approach motivation regarding an exam declines as an exam comes closer in time.

*H2b*: Students’ performance avoidance motivation regarding an exam increases as an exam comes closer in time.

In line with theoretical predictions based on temporal discounting and previous findings (e.g., König and Kleinmann, 2005; Liborius et al., 2019; Capelle et al., 2022), we expected the quantity of study activities (i.e., the time students invest into studying) to increase:

*H3*: Students’ study time increases as an exam comes closer in time.

Because previous findings regarding changes in the usage of surface and deep learning strategies over time are rather ambiguous (e.g., Zusho et al., 2003; Naujoks and Händel, 2020) and we had no firm basis to formulate theoretical expectations, we test them without assumed directionality:

*H4*: Students’ usage of learning strategies changes as the exam comes closer in time.

*H4a*: Students’ usage of surface learning strategies changes as the exam comes closer in time.

*H4b*: Students’ usage of deep learning strategies changes as the exam comes closer in time.

Furthermore, given the lack of previous research regarding the development of motivational want and should conflicts (e.g., Grund et al., 2015b; Hofer and Fries, 2016) over time, we also test them without assumed directionality:

*H5*: Students’ experience of motivational conflicts changes as the exam comes closer in time.

*H5a*: Students’ experience of want conflicts changes as the exam comes closer in time.

*H5b*: Students’ experience of should conflicts changes as the exam comes closer in time.

Finally, we were interested in how the changes in motivational and study-related variables related to one another, both with regard the apparent contradictions and potential patterns that might help to explain them. We thus considered correlations of growth factors in our models but without clear expectations.

## Methods

The present study is part of a larger project that was approved by the university’s local ethics committee before the study began. All procedures were in accordance with the ethical standards of the institutional committee and with the 1964 Helsinki declaration and its later amendments or comparable ethical standards.

### Sample and procedure

In order to test our hypotheses, we used data from a larger diary study on motivation and stress in higher education. The overall goal of the study was to track what students do and how they feel over several weeks in the preparation of a specific exam. Specifically, undergraduate students (Bachelor’s degree) from three lectures within two courses of study (education science and psychology) at a mid-sized German University were invited to participate in a weekly diary study. Education science students prepared for an exam in one of two lectures (i.e., introduction to psychology for pre-service teachers; introduction to biology didactics for pre-service teachers), while psychology students all prepared for an exam in one lecture (i.e., a statistics lecture on computer-assisted data analysis for psychology students). All lectures were structured similarly as weekly 90-min in-person sessions with one high-stakes exam at the end of the semester. Typical exams in these lectures also last 90 min and are comprised of both multiple choice and written parts.

From each lecture, approximately ¼ to ⅕ of all students agreed to participate and underwent the following procedure. First, they were invited to participate in an introduction session, where they were informed in small groups about the study rationale and reported some pre-test variables, none of which is relevant for the present contribution. Across all three lectures, 101 students completed this step. After this, for eight consecutive weeks, participants provided each Friday afternoon a retrospective diary in an online questionnaire they were invited to via a smartphone app. The assessment period started in December and ended in February. The last measurement occasion took place either three or thirteen days before the relevant exam. 96 of the initial 101 participants indicated an exam they planned to take and provided data for at least one measurement point and were therefore retained in the present analyses. Of these 96 students, 48 (50%) attended the introduction to psychology lecture, 28 (25%) attended the biology didactics lecture, and 28 (25%) attended the statistics lecture. See Table 1 for an overview of the sample statistics. Finally, we invited those participants again to provide some post-test information not relevant for the present contribution. They were thanked and received up to 40€ in compensation, depending on their compliance. They received the maximum amount for 90% or more of surveys answered and progressively less the lower their compliance (e.g., 24€ for 70%, 16€ for 60%, 8€ for 60%, and 5€ for 40%). Note that the study included six additional measurement points that were counted to determine overall compliance but are not relevant to the substance of the current study. The present study is the first publication on the larger data set.

**Table 1:** Sample statistics by study course affiliation and lecture.

Study course and lecture	*N* (% female)	Age Mean (SD)	Semester Mdn [min; max]	Measurement points (% compliance)	Days until exam [1]
**Education Science**	**72 (82%)**	**21.78 (2.93)**	**3 [1; 10]**	**473 (82.1%)**	**13 to 3**
Intro to psychology	48 (83%)	21.53 (2.13)	3 [2:10]	324 (84.4%)	13
Didactics	24 (79%)	22.25 (3.96)	5 [1; 9]	149 (77.6%)	3
**Psychology (Statistics)**	**24 (88%)**	**21.46 (2.93)**	**3 [2; 7]**	**153 (79.7%)**	**3**
**Overall**	**96 (83%)**	**21.70 (2.91)**	**3 [1; 10]**	**626 (81.5%)**	**13 to 3**

Measurements took place weekly on eight consecutive Fridays. Holidays took place between measurement points two and four. [1] After the last measurement point. Bold numbers represent values for study courses and overall sample, non-bold numbers represent values for lectures within a study course.

### Measures

We report the measures that are relevant to the current research. Other measures that were obtained during the study can be provided by the first author upon request.

#### Motivational variables

Expectancy-value beliefs (motivation quantity) were assessed using one item for expectancy (“I think I can do well on the exam.”). This operationalization was deemed adequate as the most common conceptualizations of expectancy (i.e., success expectancy and ability beliefs) are routinely assessed as one factor (Hulleman et al., 2016; Eccles and Wigfield, 2020). Similarly, attainment value was assessed with one item (“It is important to me to do well in the exam.”; *cf.* Battle, 1965; Acee et al., 2012; but see Eccles and Wigfield, 2020). Note that this operationalization has also been referred to as importance value (e.g., Crandall et al., 1962; Cole and Osterlind, 2008; Dietrich et al., 2013). As mentioned earlier, focusing on the attainment subfacet was deemed appropriate given the purpose of the present research (e.g., Perez et al., 2019). Moreover, we assumed attainment value to be reasonably representative of overall task value given its close conceptual and factorial relationship with the other subfacets of task value (e.g., Eccles and Wigfield, 1995; Jacobs et al., 2002; Durik et al., 2006; Barron and Hulleman, 2015; Lauermann et al., 2017; Parrisius et al., 2022) and utility value in particular (Zusho et al., 2003; Eccles, 2005; Musu-Gillette et al., 2015; Parrisius et al., 2022).

Similarly, in order to assess motivation quality along the performance approach-avoidance distinction (e.g., Elliot, 1999, 2006), we applied one item aiming to capture performance approach motivation (“It is important for me to do better than the other students.”) and one item capturing performance avoidance motivation (“My fear of doing poorly on the exam is what motivates me.”). All motivational variables were assessed using a six-point Likert scale on which students could indicate their agreement with the respective statements (from “does not apply at all” to “is completely true”). Note that all questions are phrased with respect to the upcoming exam as the relevant temporal landmark. Note further that single-items are commonly used in repeated measure designs (with eight measurement points in our case) in order to increase participant retention (e.g., Wanous et al., 1997) and often achieve high validity (e.g., Loo, 2002; Bergkvist and Rossiter, 2007; Allen et al., 2022).

#### Study activities

Learning time invested for the exam (study “quantity”) was assessed using the question “Approximately how much time did you spend in the last 7 days in total on the preparation and the follow-up of the lecture and the exam [lecture name], respectively, apart from accompanying seminars/exercises?.” Students could respond by indicating the number of hours they had studied during the past week. In order to assess learning strategy use (i.e., study quality) in the present study context, we adapted items from the subfacets “elaboration” of the Berlin Reading Strategy Inventory (BLSI, McElvany and Richter, 2009). Specifically, four items were rephrased so that they refer to deep learning with respect to the approaching exam (example item: “I tried to find my own examples that fit the material.”). In addition, surface learning strategies were operationalized by two items reflecting a repetitive and outcome- rather than understanding-oriented approach (example item: “I tried to memorize everything if possible.”) to learning. Both strategy complexes were treated as manifest constructs by forming the average of the responses to the four or two items, respectively. As for the motivational variables, all students could indicate their agreement with these statements on a six-point Likert scale. Note that students were only queried regarding their study strategy use when they had indicated that they had studied one hour or more (i.e., when their response to the study quantity question was >0). Consequently, we recorded fewer measurement points for learning strategies than for the other constructs (*cf.* Table 2).

**Table 2:** Descriptive statistics and correlations.

	n	*M*	*SD*	Min	Max	1	2	3	4	5	6	7	8	9	10
1. Measurement Point	768	4.500	2.309	1	8	--									
2. Attainment Value	626	3.899	1.210	0	5	−0.093	--								
3. Expectancy	626	3.134	0.974	0	5	−0.102	0.272	--							
4. Perf. Approach	626	1.705	1.642	0	5	−0.034	0.438	0.146	--						
5. Perf. Avoidance	626	2.393	1.534	0	5	0.075	0.354	−0.127	0.424	--					
6. Study Time	626	3.788	4.643	0	40	0.360	0.109	0.085	0.025	0.037	--				
7. Surface Learning	498	2.845	1.197	0	5	0.363	0.006	−0.075	0.027	0.137	0.194	--			
8. Deep Learning	498	2.807	1.085	0	5	0.214	−0.069	0.145	−0.021	−0.031	0.164	0.378	--		
9. Want Conflict	626	2.430	1.388	0	5	0.117	0.066	−0.190	0.110	0.233	0.026	0.102	0.040	--	
10. Should Conflict	626	2.621	1.362	0	5	−0.096	0.100	−0.138	0.140	0.245	−0.200	0.047	−0.054	0.444	--

Descriptive statistics and correlations refer to variables over all eight measurement points and ignore the nested data structure.

#### Motivational conflicts

Following Grund et al. (2015a), participants indicated their experience of motivational conflicts over the past seven days by answering the question “Within the last 7 days, to what extent did you feel, overall, that you rather wanted to do something other than what you actually did?” to indicate want-conflicts and “…that you should rather be doing something other than what you actually did?” to indicate should-conflicts on a six-point Likert scale from “not at all” to “very much.”

#### Control variables

In order to account for potential differences between groups of students, we considered four control variables in an additional modeling step (see below). Exams were written on two separate dates: The didacts exam (education science) and the statistics exam (psychology) were written three days after the last measurement point while the introduction to psychology (education science) exam was written ten days later (*cf.* Table 1). We consequently considered the exam date as a control variable (0 = earlier, 1 = later; Mehta and West, 2000). Additionally, we considered study course (0 = education science, 1 = psychology). Note that taken together, the dummy variables for exam date and study course distinguish all three lectures and their respective exams in our sample (e.g., Didactics: exam date = 0; study course = 0; *cf.* Table 1). Additionally, we considered students’ age and gender (0 = male, 1 = female).

### Analytical procedure

#### Statistical modeling

We used unconditional parallel process models, that is, first order latent growth curve models, to statistically model the trajectories of our focal variables among all students in our sample (e.g., Geiser, 2012; see Kosovich et al., 2017, and Robinson et al., 2019, for similar approaches). In order to model the passage of time, the eight measurement points were used as factor weights for each measurement point, starting at zero, thus setting the model intercepts at the beginning of the eight-week time period covered (Geiser, 2012). We reasoned that setting intercepts at the beginning of the assessment period to be most informative as it allowed us to consider the correlation of students’ initial motivation and study behavior with changes over time. We tested a parallel process model concerned with motivation (four variables), study activities (three variables), and motivational conflicts (two variables) separately. In order to account for potential differences in growth factors between individuals, we subsequently regressed all growth factors in each model on the four control variables (conditional parallel process models). Statistical significance tests were one-sided for directed hypotheses (i.e., H2 and H3) and two-sided for all undirected hypotheses as well as the control variables in the conditional growth models.

#### Missings and dropout analysis

Participants answered 81.5% of all weekly survey invitations (626 out of 776). We plotted the missing patterns for all variables and visually screened them for systematic dropout over time. We could not find any apparent evidence of systematic dropout. In case of the motivational variables, the average percentage of missings per measurement point was 19.2% and varied between a minimum of 13.4% on the first measurement point and a maximum of 22.7% on the third measurement point. In case of the variables concerned with learning strategies, which students were only asked about if they indicated at least one hour of studying in the previous week, 64.3% of measurement points were answered (499 out of 776). Here, the average of missings per measurement point was 32.8% and varied between a minimum of 25.8% on the last two measurement points and a maximum of 61.9% on the third measurement point. The relatively high missing rates on the third measurement point can likely be attributed to the fact that it occurred during holidays between Christmas and New Year, which occurred between measurement points two and four. Missings were handled using the Full Information Maximum Likelihood (FIML) method. Note that the final sample size (*N* = 96 individuals with 626 and 776 measurement points, respectively) is well within the recommended range of sample sizes assumed to yield reliable results (Muthén and Curran, 1997; Maas and Hox, 2005; Curran et al., 2010).

## Results

### Descriptive results

Table 2 summarizes the overall descriptive statistics of all variables and the overall correlations between all measures, ignoring the data structure (i.e., measurements nested within individuals).

### Inferential results

As our research interest was mainly the change of students’ motivation and study activities over time, we focus the narrative presentation of our results mainly on the linear growth factors (i.e., slopes) of the respective variables. Following Kosovich et al. (2017), we additionally report variance of growth factors and correlations between growth factors.

#### Motivation

**Figure 1:**
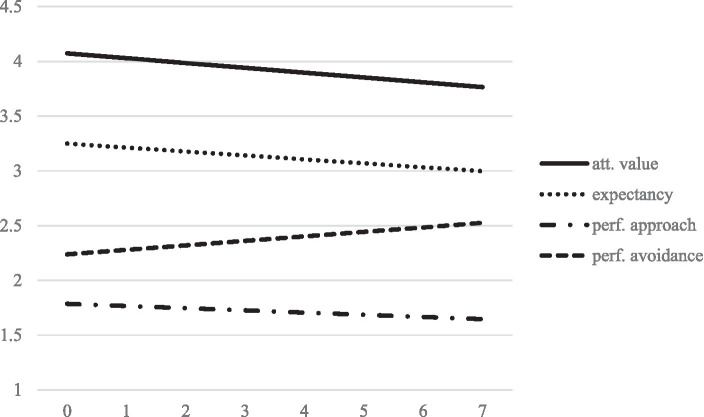
Model implied linear trajectories of motivational variables over measurement points.

We first modeled the trajectories of the four motivational variables. The results are summarized in Table 3. Model-implied trajectories are shown in Figure 1. The linear growth factors of expectancy (*p* = 0.031) and attainment value (*p* = 0.015) beliefs are statistically significant and indicate a decline in both constructs over time for the average student. We therefore retain H1. The slope of performance approach motivation is not statistically significant (*p* = 0.118) but its estimated coefficient pointed toward an average decline. In contrast, performance avoidance motivation increases statistically significantly (*p* = 0.016) over time. We thus partially retain H2. All growth factors are characterized by statistically significant variance between individuals. Moreover, the slopes of expectancy and value (*p* = 0.008), expectancy and performance approach motivation (*p* = 0.012), and attainment value and performance approach motivation (*p* = 0.002) are statistically significantly and positively correlated, indicating that their decline occurred in tandem for many students. Moreover, the slopes of performance-approach and performance avoidance were positively correlated (*p* = 0.048), indicating that students whose performance avoidance motivation increases more steeply, performance approach motivation decreases less steeply or even increases over time.

**Table 3:** Motivational variables unconditional growth factor estimates.

		Estimate	Variance	95% Confidence Interval		Correlations							
				LL	UL	1	2	3	4	5	6	7	8
1	Value Intercept	4.073***	0.804***	3.873	4.273	--							
2	Att. Value Slope	−0.044*	0.018***	−0.079	−0.009	−0.075	--						
3	Expectancy Intercept	3.249***	0.632***	3.072	3.426	0.242*	0.007	--					
4	Expectancy Slope	−0.036*	0.013***	−0.068	−0.004	0.020	0.492**	−0.306*	--				
5	Perf. Appr. Intercept	1.786***	2.159***	1.477	2.096	0.538***	0.049	0.138	−0.011	--			
6	Perf. Appr. Slope	−0.020	0.015***	−0.054	0.013	−0.094	0.572***	0.101	0.472**	−0.043	--		
7	Perf. Avo. Intercept	2.238***	1.219***	1.986	2.490	0.391**	0.048	−0.271*	0.040	0.461***	−0.075	--	
8	Perf. Avo. Slope	0.041*	0.013*	0.004	0.078	0.282	0.251	0.148	−0.044	0.228	0.435*	0.275	--

Att., attainment; Perf. Appr., performance approach; Perf. Avo, performance avoidance; *p < 0.05; **p < 0.01; ***p < 0.001; Χ^2^/df = 1.793; RMSEA = 0.091; CFI = 0.857; TLI = 0.854; SRMR = 0.074.

#### Study activities

**Figure 2:**
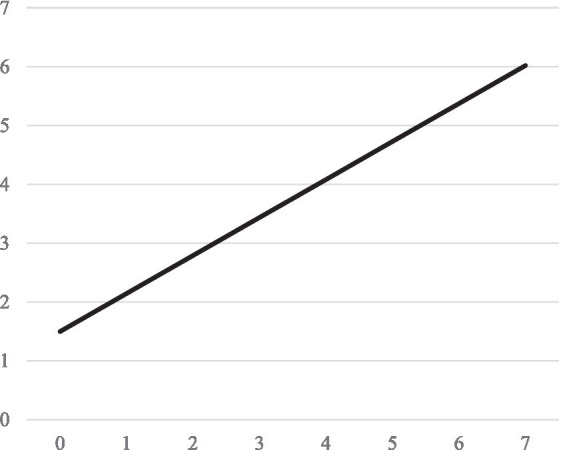
Model implied linear trajectory of time spent studying in hours over measurement points.

**Figure 3:**
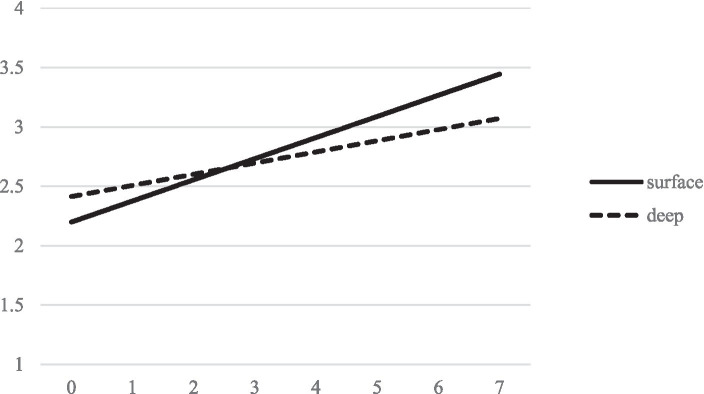
Model implied linear trajectories of the use of surface and deep learning strategies over measurement points.

The second model is concerned with study activities. Results are summarized in Table 4 and model-implied trajectories are shown in Figures 2, 3, respectively. The growth factor for study quantity indicates that study time increased significantly (*p* < 0.001). Specifically, the result indicates that the average student in our sample studied approximately 1 h and 30 min per week for the exam at the beginning of the sampling period and around 6 h and 40 min per week at the last measurement point. We therefore retain H3. With regard to learning strategies, results indicate that both surface learning strategy usage (*p* < 0.001) and deep learning strategy usage (*p* < 0.001) increase over time. However, the growth of surface learning strategies was substantially larger than the growth of deep learning strategies, indicating that students tended to use more surface learning strategies than deep learning strategies as the exam comes closer. We therefore retain H4.

**Table 4:** Study activity variables unconditional growth factor estimates.

		Estimate	Variance	95% Confidence Interval		Correlations					
				LL	UL	1	2	3	4	5	6
1	Surface Intercept	2.199***	0.825***	1.973	2.426	--					
2	Surface Slope	0.178***	0.013**	0.142	0.214	−0.421*	--				
3	Deep Intercept	2.414***	1.012***	2.188	2.639	0.584***	−0.397*	--			
4	Deep Slope	0.094***	0.011**	0.064	0.124	−0.468**	0.701***	−0.522***	--		
5	Study time Intercept	1.518***	0.757	1.119	1.917	0.176	−0.042	0.421	−0.306	--	
6	Study time Slope	0.644***	0.601***	0.449	0.839	−0.170	0.350	0.009	0.081	−0.372	--

Surface, surface learning strategy use; deep, deep learning strategy use; *p < 0.05; **p < 0.01; ***p < 0.001. *Χ*^2^/df = 2.983. RMSEA = 0.144. CFI = 0.552. TLI = 0.547. SRMR = 0.149.

With regard to the correlations of growth factors, results indicate that the slopes of surface and deep learning strategies are positively correlated (*p* = 0.004). Interestingly, the intercept and slope of both learning strategies (surface: *p* = 0.012; deep: *p* < 0.001) are negatively correlated, indicating that students who used a learning strategy less at the beginning of the sampling period tended to increase the use of that strategy more rapidly as the exam approached. Likewise, while the intercepts of deep and shallow learning strategy use are positively correlated with each other (*p* < 0.001), they are each negatively correlated with the slope growth factors of the respective other construct. This means that students who tended to use more deep learning strategies at the beginning of the sampling period tended to increase their use of shallow learning strategies less steeply (and vice versa). Moreover, variance of all growth factors except for the study time intercept was statistically significant, indicating that students’ study time was similarly low at the beginning of the sampling period.

#### Motivational conflicts

**Figure 4:**
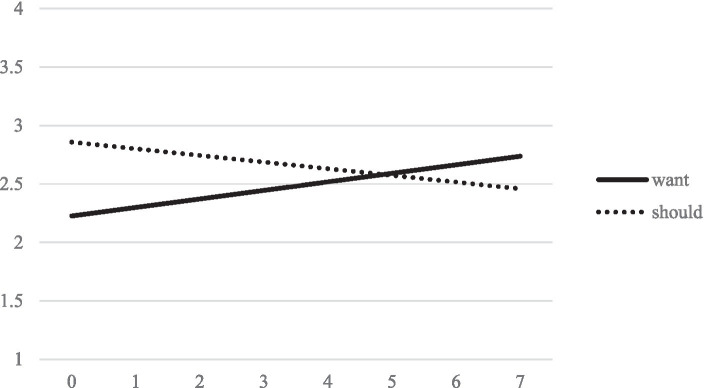
Model implied linear trajectories of want and should conflicts over measurement points.

The third model complements the previous analyses by considering the changes of want and should conflicts over the time period. Model results are summarized in Table 5 and model-implied trajectories are shown in Figure 4. Results indicate that want conflicts increased over time (*p* = 0.002). In contrast, should-conflicts decreased over time (*p* = 0.007). We therefore retain H5.

**Table 5:** Motivational conflict variables unconditional growth factor estimates.

		Estimate	Variance	95% Confidence Interval		Correlations			
				LL	UL	1	2	3	4
1	Want Intercept	2.226***	0.418*	2.010	2.442	--			
2	Want Slope	0.073**	0.017*	0.027	0.119	−0.010	--		
3	Should Intercept	2.858***	0.639**	2.632	3.084	0.911***	0.044	--	
4	Should Slope	−0.057**	0.008	−0.099	−0.016	0.122	0.418	−0.171	--

Want = Experience of want conflicts; Should = experience of should conflicts; *p < 0.05; **p < 0.01; ***p < 0.001. Χ^2^/df = 1.417. RMSEA = 0.066. CFI = 0.856. TLI = 0.858. SRMR = 0.095.

Interestingly, the intercepts of want and should conflicts were strongly positively correlated (*p* < 0.001), indicating that students who experienced more want conflicts at the beginning of the sampling period also experienced more should conflicts and vice versa. Moreover, results indicate statistically significant variance between students for all growth factors except the slope for should conflicts, indicating that the decline in should conflicts over the sampling period was fairly universal among students.

#### Conditional growth models: individual differences

In order to account for potential differences in growth factors between students, we introduced the control variables age, study course, gender, and exam date (early vs. late) into the three unconditional models.

With regard to motivation, results show that the intercepts of both attainment value (*b* = 1.089, *SE* = 0.255, *p* < 0.001) and performance approach motivation (*b* = 0.834, *SE* = 0.419, *p* = 0.047) varied with study course affiliation. Specifically, both were higher for psychology students at the beginning of the assessment period. Moreover, the linear slope growth factor of expectancy varied significantly with age (*b* = −0.018, *SE* = 0.006, *p* < 0.001). Specifically, the younger students are, the more their expectancy declines as the exam comes closer. Finally, the intercept of performance-avoidance motivation varied with age (*b* = 0.098, *SE* = 0.044, *p* = 0.027). Specifically, the older students are, the higher were their initial performance avoidance motivation at the beginning of the sampling period.

With regard to study activities, results show that the linear slope growth of study quantity varied with age (*b* = −0.084, *SE* = 0.028, *p* = 0.003), indicating that younger students increased their study activities less rapidly than older students. The study quantity slope also varied with study course affiliation (*b* = 1.108, *SE* = 0.219, *p* < 0.001), indicating that psychology students increased their study efforts more rapidly over time. With regard to learning strategies, results show that the intercept of shallow learning strategies varied with study course affiliation (*b* = −0.600, *SE* = 0.028, *p* = 0.049), indicating that psychology students used fewer shallow learning strategies at the beginning of the assessment period. Moreover, results show that age predicted variance of deep learning strategies both with regard to intercepts (*b* = 0.115, *SE* = 0.036, *p* = 0.001) and slopes (*b* = −0.016, *SE* = 0.005, *p* = 0.002). Specifically, younger students used more deep learning strategies at the beginning of the assessment period but increased their use less rapidly.

With regard to conflict experience, results show that the intercepts of should conflicts varied with gender (*b* = 0.712, *SE* = 0.305, *p* = 0.020), indicating that female students experienced more should conflicts at the beginning of the assessment period. Moreover, the linear slopes of should conflicts varied with study course affiliation (*b* = 0.137, *SE* = 0.056, *p* = 0.015), indicating that should conflicts decreased less and even increased among psychology students.

#### Post-hoc analysis: non-linear models

**Figure 5:**
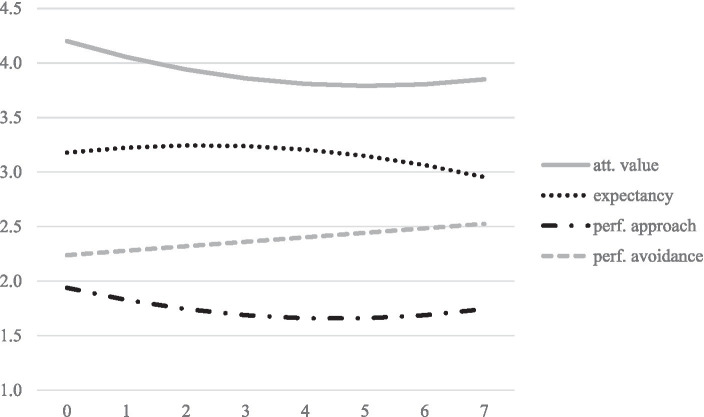
Model implied nonlinear trajectories motivational variables. The trajectories were selected by best model fit and each represent one individual model. Trajectories of value and performance-avoidance (grey) were statistically non-significant.

**Figure 6:**
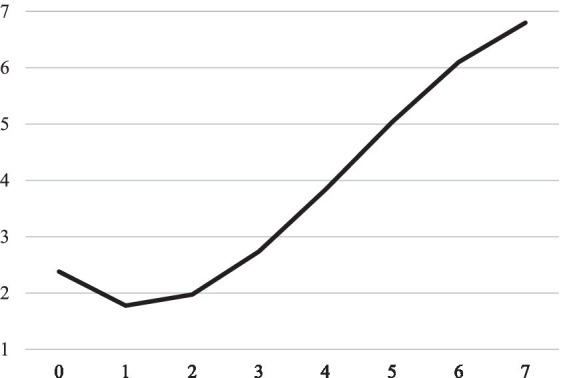
Model implied nonlinear trajectories of time spent studying in hours based on best model-fit.

Having conducted the main analyses, we noticed that the model fit was not very good for the models concerned with study activities (*cf.* Table 4). This is not very surprising given the high number of parallel processes and that the Christmas holidays were included in the sampling period between measurement points two and four, which is likely to interfere with any study routine, as we discuss below. Still, we were interested in how the linear trajectories we investigated compared to nonlinear growth models. Specifically, we were interested if nonlinear models might provide more nuanced insights into how our central variables developed over time for future research. We thus compared the linear growth models of single variables (rather than the full unconditional parallel process models we reported above) to models including a quadratic and cubic growth factor based on common relative fit indices (AIC and BIC) while considering absolute indices of model fit (see Table 6). We found that linear models fit best for performance avoidance motivation, surface and deep learning strategies as well as want and should conflicts. We further found that quadratic models best fit for expectancy, expectancy, and performance approach motivation. However, quadratic growth factors were not statistically significant for value and performance approach motivation. Best fit with an additional cubic growth factor was found for study quantity. While these additional growth factors can provide additional nuance, the central messages that can be derived from the linear models stay the same (see Figures 5, 6 for model-implied trajectories of the non-linear models).

**Table 6:** Fit comparisons of models with single variables.

Variable	Fit index	Intercept only	Linear slope	Quadratic slope	Cubic slope
Attainment value	AIC	1603.567	1540.398	**1511.582**	
	BIC	1629.211	1573.734	**1555.176**	
	Adjusted BIC	1597.636	1532.688	**1501.50**	
	Χ^2^	151.45***	82.281***	45.465*	
	Χ^2^/df	4.454	2.654	**1.684**	
	RMSEA	0.189***	0.131***	**0.084**	
	CFI	0.779	0.904	**0.965**	
	TLI	0.818	0.913	**0.964**	
	SRMR	0.213	0.121	**0.076**	
Expectancy	AIC	1417.327	1381.707	**1380.265**	
	BIC	1442.970	1415.043	**1423.858**	
	Adjusted BIC	1411.396	1373.997	**1370.182**	
	Χ^2^	81.812***	40.192	**30.750**	
	Χ^2^/df	2.406	1.297	**1.139**	
	RMSEA	0.121**	0.056	**0.038**	
	CFI	0.870	0.975	**0.990**	
	TLI	0.893	0.977	**0.989**	
	SRMR	0.099	0.061	**0.061**	
Performance approach	AIC	1591.801	1559.196	**1554.374**	
	BIC	1617.445	1592.532	**1597.968**	
	Adjusted BIC	1585.871	1551.485	**1544.292**	
	Χ^2^	117.319***	78.713***	65.891***	
	Χ^2^/df	3.451	2.539	**2.440**	
	RMSEA	0.160***	0.127***	0.122**	
	CFI	0.908	0.947	**0.957**	
	TLI	0.924	0.953	**0.956**	
	SRMR	0.066	0.052	**0.043**	
Performance avoidance	AIC	1840.888	**1815.957**	1821.396	
	BIC	1866.532	**1849.294**	1864.990	
	Adjusted BIC	1834.958	**1808.247**	1811.313	
	Χ^2^	74.255***	**43.324**	40.762	
	Χ^2^/df	2.184	**1.398**	1.510	
	RMSEA	0.111**	**0.064**	0.073	
	CFI	0.923	**0.976**	0.974	
	TLI	0.937	**0.979**	0.973	
	SRMR	0.102	**0.051**	0.048	
Want conflict	AIC	2100.765	**2080.519**	2083.545	
	BIC	2126.408	**2113.855**	2127.139	
	Adjusted BIC	2094.834	**2072.808**	2073.463	
	Χ^2^	67.607***	**41.360**	36.387	
	Χ^2^/df	1.988	**1.334**	1.348	
	RMSEA	0.101*	**0.059**	0.060	
	CFI	0.728	**0.916**	0.924	
	TLI	0.776	**0.924**	0.921	
	SRMR	0.124	**0.091**	0.080	
Should conflict	AIC	2056.646	**2050.845**	2056.918	
	BIC	2082.289	**2084.181**	2100.512	
	Adjusted BIC	2050.715	**2043.134**	2046.835	
	Χ^2^	49.690*	**37.889**	35.962	
	Χ^2^/df	1.461	**1.222**	1.332	
	RMSEA	0.069	**0.048**	0.059	
	CFI	0.883	**0.949**	0.933	
	TLI	0.904	**0.954**	0.931	
	SRMR	0.108	**0.087**	0.084	
Surface learning	AIC	1452.077	**1332.467**	1335.083	
	BIC	1477.51	**1365.53**	1378.319	
	Adjusted BIC	1445.94	**1324.489**	1324.65	
	Χ^2^	174.725***	49.116*	43.732*	
	Χ^2^/df	5.139	**1.584**	1.620	
	RMSEA	0.210***	**0.079**	0.081	
	CFI	0.376	**0.920**	0.926	
	TLI	0.486	**0.927**	0.923	
	SRMR	0.231	**0.14**	0.137	
Deep learning	AIC	1206.155	**1137.840**	1144.079	
	BIC	1231.588	**1170.903**	1187.315	
	Adjusted BIC	1200.018	**1129.862**	1133.646	
	Χ^2^	116.290***	**41.975**	40.214*	
	Χ^2^/df	3.420	**1.354**	1.489	
	RMSEA	0.160***	**0.061**	0.072	
	CFI	0.770	**0.969**	0.963	
	TLI	0.811	**0.972**	0.962	
	SRMR	0.140	**0.094**	0.094	
Study time	AIC	3468.216	3327.030	3283.529	**3260.242**
	BIC	3493.860	3360.367	3327.123	**3316.657**
	Adjusted BIC	3462.286	3319.320	3273.447	**3247.194**
	Χ^2^	286.578***	139.392***	87.891***	54.603***
	Χ^2^/df	8.429	4.497	3.255	**2.482**
	RMSEA	0.278***	0.191***	0.153***	0.124**
	CFI	0.000	0.439	0.685	**0.831**
	TLI	0.000	0.493	0.673	**0.785**
	SRMR	0.322	0.234	0.130	**0.095**

AIC, Akaike Information Criterion; BIC, Bayesian Information Criterion; Adjusted BIC, Sample-Size Adjusted Bayesian Information Criterion; Χ^2^, Chi-Square Test of Model Fit; *Χ*^2^/df, Chi-Square Test of Model fit divided by Degrees of Freedom; RMSEA, Root Mean Square Error of Approximation; CFI, Comparative Fit Index; TLI, Tucker-Lewis Index; SRMR, Standardized Root Mean Square Residual. Best-Fitting model is highlighted in bold. *p < 0.05; **p < 0.01; ***p < 0.001.

## Discussion

### Summary and interpretation of findings

In the present research, we set out to answer two central questions. First, how study motivation and study behavior change as an exam comes closer in time? Secondly, do changes in directly measured study motivation covary systematically and positively with changes in study behavior? We asked the first question against the background of recently resurgent interest in the role of contextual factors shaping situationally varying motivation, we identified upcoming temporal landmarks (such as exams) as candidate context factors likely to help explain systematic variations in motivation. In order to derive the second question, we integrated findings from largely distinct parts of the literature (*cf.* Steel and König, 2006; Steel and Weinhardt, 2018): The decline in expectancy and value beliefs (i.e., motivation quantity) over time has mainly been found in educational psychology (e.g., Zusho et al., 2003; Kosovich et al., 2017; Benden and Lauermann, 2022), while increases in study quantity over time has mainly been found in behavioral economics research (e.g., Dewitte and Schouwenburg, 2002; König and Kleinmann, 2005; Liborius et al., 2019). Our first avenue for clarifying these apparent differences was to consider the trajectories of motivation quantity and study quantity on equal methodological terms (i.e., over the same time period, with the same specificity of measurement, and reference to the same temporal landmark). In addition, we considered the trajectories of approach and avoidance motivation as measures of motivation quality, the use of deep and surface learning strategies, and motivational conflicts as indicators of latent study motivation.

Interestingly, our results suggest that both expectancy and attainment value with respect to the exam declined (H1) while study time (study quantity) increased as exams came closer (H3). It therefore seems that while the temporal landmarks seem to account for systematic changes in the quantity of both study motivation and study behavior, methodological alignments cannot by themselves reconcile their trajectories: In concordance with previous findings, study quantity but not motivation quantity changed in accordance with the predictions of temporal discounting. In this sense, the trajectory of approach and avoidance motivation offer additional insights. While performance approach motivation tended toward a decline but did not change significantly, performance avoidance motivation increased as the exam came closer (H2). These findings are partially in accordance with conflict theories (e.g., Trope and Liberman, 2003) and seem compatible with previous findings that associated lower expectancies with higher avoidance goal adoption (Elliot and Church, 1997; Senko, 2016). Importantly, they suggest that the increase in study activities in our sample was primarily driven by avoidance motivation. Put bluntly: students are indeed more “engaged” when exams come closer – which indicates an increase in motivation – but they are because they fear negative consequences. We discuss this as well as other possible explanations for our findings in the context of recent theoretical discourse below. One reason why performance approach goals might not have changed significantly is that, in contrast to the conception of approach tendencies as being entirely appetitive in older conflict theories, performance approach goals have been shown have both appetitive and aversive antecedents and consequences (Carver, 2006; Warburton and Spray, 2009; Senko, 2016).

Our results further indicate that the use of both surface and deep learning strategies increased as the exams approached (H4). However, surface learning strategies increased substantially stronger than deep learning strategies (*cf.* Figure 3). We thus interpret these increases as partially mixed with increases in study quantity: As students increased their studying over time, their use of study strategies necessarily increased. As the use of shallow learning strategies surpasses the use of deep learning strategies between the second and third measurement point, the results can be tentatively interpreted as an overall decrease in the quality of study activities. As was discussed earlier, this change in students’ choice of learning strategies can be seen as an adaptive choice of learning strategies before the exam: As the exam comes closer, students might increasingly switch to surface learning strategies to consolidate the knowledge they have previously acquired using deep learning strategies. Moreover, shallow and short-term knowledge of a topic is often both necessary and sufficient to pass an exam (Busato et al., 1998; Vermunt, 1998; Berger and Karabenick, 2011). Students might become increasingly concerned with passing the exam and switch to surface learning strategies in order to ensure that they pass. Indeed, this interpretation seems compatible with the increase in performance avoidance motivation which has been linked to the use of surface learning strategies (e.g., Duncan and McKeachie, 2005; Liem et al., 2008; *cf.* Elliot, 1999). Finally, our results indicate that students’ experience of should-conflicts decreases over time while want-conflicts increase. This again seems broadly compatible with our other findings: Although we did not specifically ask students what activities they felt conflicted about, previous research indicates that many of the should-conflicts that students experience are likely concerned with neglecting studying in favor of doing something else (Grund et al., 2014; Capelle et al., 2022). As students increased their study activities over time, they hence experienced fewer should-conflicts (i.e., they increasingly did what they “should” do). At the same time, their increasing study activities likely came at the cost of other goals and needs, hence increasing students’ want-conflicts. In addition, want-conflicts could be facilitated as students tire of their studying and possibly amplified if their studying was increasingly driven by avoidance motivation (Grund et al., 2014). Interestingly, this tentatively suggests that the kind of opportunity costs that students experience during an exam period changes as the exam draws closer (e.g., Flake et al., 2015; Emanuel et al., 2022).

Finally, with regard to interindividual differences, the main differences between students in our sample seem to be concerned with age and study course affiliation (education science vs. psychology). Indeed, higher initial avoidance motivation and use of deep learning strategies among older students as well as stronger declines in expectancy as well as slower increases in the use of study quantity and deep learning strategy use among younger students seem broadly compatible with a view that older students have had more opportunity to become more adept with exams in a university setting. Regarding study course affiliation, higher initial value beliefs, approach motivation, and lower surface learning strategy use as well as stronger increases in study quantity and lower decreases (and even increases) in should conflicts among psychology students might reflect that psychology programs, at least in Germany, are more selective than most other programs based on high school grades and thus achievement-related personality traits (e.g., Ziegler et al., 2009).

### Theoretical implications

Fundamentally, our results show that the approach of temporal landmarks such as an exam can explain systematic changes in situational motivation and study behavior among university students. As such, one of the main contributions of the present research is to highlight the role that temporal landmarks play to better understand the dynamics of study motivation and study behavior. While we did not assess the entirety of relevant constructs of one particular theory, the present research has potentially important implications for research concerned with expectancy-value theories such as Situated Expectancy-Value Theory (Eccles and Wigfield, 2020) and achievement goal theories such as the hierarchical model of approach and avoidance achievement motivation (Elliot and Church, 1997) or the 2 × 2 achievement goal framework (Elliot and McGregor, 2001).

Methodologically, our findings suggest that the time of measurement of motivation might be crucial, even if the research is not concerned with temporal landmarks *per se* (for similar arguments, see Senko and Harackiewicz, 2005). For example, students’ overall level of motivation might depend, at least in part on how close a relevant temporal landmark is, although it remains to be seen whether and to what extent task-specific deadline-effects can be observed at higher levels of measurement specificity (e.g., class- or course-specific; *cf.* Baranik et al., 2010; Richey et al., 2018).

Substantively, temporal landmarks are evidently a contextual factor that can account for systematic changes in study motivation and study behavior. While it is clear that both expectancy-value beliefs and achievement goals are to some degree context-dependent and thus vary within persons (e.g., Hulleman et al., 2016; Dietrich et al., 2019), the contextual factors contributing to these variations have yet to be fully understood. For example, systematic situational changes have so far mainly been observed after or between performance tests such as exams or assignment deadlines (e.g., Fryer and Elliot, 2007; Muis and Edwards, 2009; Benden and Lauermann, 2022). As such, changes in motivation in anticipation of a performance test offer a meaningful, complimentary perspective. Indeed, given our as well as previous findings, temporal landmarks might be considered “strong” situation at least with respect to the fairly universal increases in study behavior (Taraban et al., 1999; König and Kleinmann, 2005; Peetz and Wilson, 2013; Dweck, 2017; Liborius et al., 2019; Capelle et al., 2022).

Theoretically, it remains to be seen whether temporal landmarks should find theoretical consideration in models of achievement motivation (for example, in the “right side” of the Situated Expectancy Value model which is concerned with the most proximal predictors of motivation; Eccles and Wigfield, 2020). Specifically, future research might consider changes in a full set of constructs of a theory in order to determine whether the distance of a temporal landmark explains enough variance to outweigh considerations of parsimony. Similarly, future research might determine whether temporal discounting more generally and predictions by Temporal Motivation Theory or conflict theories more specifically is a useful theoretical mechanism underlying temporal changes in students’ motivation and study behavior. Another important theoretical contribution of the present research pertains to the situational relationship between motivational beliefs and actual study behavior: Addressing and explaining the apparent contradiction between declining expectancy-value beliefs and increasing study time beyond methodological considerations could yield important theoretical insights. Thus, we now offer some approaches that might explain our findings and could guide future research.

#### Motivational beliefs at different phases of goal pursuit

One way to make sense of our findings is to consider more closely the different contributions expectancy and value might have at energizing study behavior at different phases of goal pursuit (Liberman and Förster, 2008; Vancouver et al., 2008; Steel and Weinhardt, 2018). According to this line of thought, high levels of expectancy and value are not necessarily positively related to study activities, depending on the time point in the goal pursuit process. Different stages in goal pursuit have long been proposed by different theories (e.g., Lewin et al., 1944; Heckhausen and Gollwitzer, 1987; Vancouver et al., 2008; for an overview, see Steel and Weinhardt, 2018). While different goal phases are not the focus of the present research, earlier stages of goal pursuit likely correspond to greater temporal distances from a temporal landmark while later stages likely correspond to smaller temporal distances. As such, they are worth considering to make sense of our results. A major difference that has been discussed in the literature is between goal choice (e.g., deciding to take an exam) and actual goal pursuit (e.g., investing time and resources into studying), which will guide our discussion.

#### Expectancy component

The idea of different stages of goal pursuit is especially applicable to the expectancy component, as the relationship of expectancy beliefs and study behavior may change at different stages of goal pursuit: In contrast to the generally positive association of expectancy beliefs and performance, some research has found negative relationships of expectancy and performance or effort in various contexts (Vancouver et al., 2001; *cf.*: Bandura and Locke, 2003; for an overview, see Vancouver et al., 2008). A possible solution to these seemingly disparate findings is the assumption of a nonmonotonic relationship of expectancy and effort, depending on the stage of the goal process (Kukla, 1972; Vancouver et al., 2008). According to this line of thought, expectancy beliefs are important to determine whether someone decides to pursue or abandon a goal early in the goal pursuit process (i.e., the goal choice phase): if someone has high expectancy beliefs with regard to a sufficiently valuable goal (e.g., passing an exam), then they are likely to pursue the goal (i.e., start studying), while someone with low expectancy beliefs deems a goal unachievable and thus likely abandons it (Kukla, 1972; Gollwitzer, 1996; Vancouver et al., 2008). Indeed, it has been argued that an “optimism bias,” the tendency to underestimate the effort required to achieve a certain goal, facilitates goal commitment at this stage (Sharot, 2011; Steel and Weinhardt, 2018). Nonmonotic models further assume that during actual goal pursuit, lower expectancies predict greater (study) effort (Vancouver et al., 2008; Steel and Weinhardt, 2018). Steel and Weinhardt (2018), referencing Bandura (1997) suggest: “in a classroom setting, where individuals need to prepare for an upcoming exam, low rather than high self-efficacy may be preferred” (p. 11). Self-efficacy describes an individual’s perceived ability to perform a specific task and thus similar but not equal to most measures of expectancy beliefs (Hulleman et al., 2016). According to this line of thought, the decline in expectancy beliefs and increase in study behavior over time we and others found might thus reflect high expectancy beliefs at the stage of goal choice at the beginning of the sampling period when not much studying takes place. Decreasing expectancies during goal pursuit might then reflect, for example, that students realize how much they still have left to study. This realization would, however, indeed be necessary to energize increasing study efforts. As mentioned, this idea is compatible with the interpretation offered above that students tend to study out of fear close to the exams (and as indicated by increasing performance avoidance motivation), which we will discuss in more detail below.

Contrary to this line of reasoning, however, Liberman and Förster (2008) describe the “‘cold feet’ phenomenon,” according to which a task may seem subjectively more difficult and less controllable (hence lowering expectancy) as it comes closer, leading to decreased motivation (see also Gilovich et al., 1993; Savitsky et al., 1998). One approach that might help to reconcile these somewhat contradictory assumptions regarding expectancy and its relationship with study effort over time is to consider different sub-facets of the expectancy construct than are typically considered in educational expectancy-value research. Indeed, Liberman and Förster (2008), largely reflecting Heckhausen and Rheinberg’s (1980) “extended cognitive motivation model“, distinguish between four different facets of expectancy that may change differently as a deadline approaches (for similar considerations see Liberman and Trope, 1998): (i) subjective task difficulty, which may either remain constant or increase (as per the “cold feet” phenomenon mentioned above), (ii) sufficiency, describing the “probability of passing [an exam] given studying” (p. 518), thus entailing both self-efficacy (e.g., Bandura, 1997) and controllability (e.g., Rotter, 1966), (iii) necessity, describing “the likelihood of achieving the goal without the action” (Liberman and Förster, 2008, p. 518), and (iv) probability, reflecting the uncontrollable subjective probability of some event to occur, which seems less applicable to an exam. The authors describe different situations in which each of these types of expectancy may either increase, remain constant or decrease as an exam comes closer under different circumstances (Liberman and Förster, 2008, p. 524), and are thus not readily applicable to explain previous findings. Nonetheless, the distinctions seem promising to gain more fine-grained insights into the trajectory of expectancy and its relation to studying in future research. For example, our operationalization of expectancy in the present study mainly reflects what Liberman and Förster (2008) call “sufficiency.” Future research could consider these subfacets of expectancy (possibly in addition to the more commonly used success expectancy and ability beliefs) in order to examine their changes and differential contribution to study behavior over time. At the same time, there is a risk that the relationship of motivational beliefs and study activities becomes theoretically arbitrary if predictions are not sufficiently precise and thus theoretically dissatisfying. It therefore seems necessary to formulate and to test exact “boundary conditions” for when which sub-facets of expectancy are predicted to be positively or negatively related to actual study behavior.

#### Value component

With regard to value, temporal discounting predicts that subjective value of studying (in our case, attainment or importance value) should increase as an exam comes closer in time and thus motivate studying (Ainslie, 1992; Schouwenburg and Groenewoud, 2001; Frederick et al., 2002; Steel and König, 2006). Why then do we, as have others, observe decreasing (attainment) value and increasing study activities as an exam comes closer? Again, the idea of different stages of goal pursuit seems helpful. In the following, we discuss two lines of thought: (i) changes in motivation quality as the valence of the value component and (ii) explicit devaluation as a protective mechanism against exam-related anxiety.

##### Change in motivation quality over time

A promising avenue of inquiry is the division of the value component into different motivation qualities along the approach-avoidance distinction and their change over time (Trope and Liberman, 2003; Steel and Weinhardt, 2018), possibly in addition to more commonly used subfacets of value (i.e., intrinsic, attainment, utility, and cost value; Eccles and Wigfield, 2020; Elliot, 2023). The trajectories of performance approach and performance avoidance motivation we found are partially in line with predictions of conflict theories (e.g., Dollard and Miller, 1950), adding to previous findings from consumer research (Mogilner et al., 2007). Recall that conflict theories assume that avoidance goals are discounted more steeply than approach goals. Accordingly, (performance-) avoidance motivation is expected to be more salient and relevant for study behavior than (performance-) approach motivation the closer the exam gets in time. As mentioned, one possible way to make sense of increasing study activities in the context of declining value-based motivation is to assume that studying close to an exam is primarily driven by avoidance motivation. Indeed, Temporal Motivation Theory (Steel and Weinhardt, 2018) explicitly accounts for this possibility with reference to Prospect Theory (Tversky and Kahneman, 1992). According to this line of reasoning, any avoidance-related motivation is more likely to energize actual behavior, at least in the short term. At least in the context of an upcoming exam, performance avoidance motivation might thus be a better situational predictor of students’ study efforts than other forms of motivation, despite its general association with worse outcomes (Hulleman et al., 2016; Senko, 2016; Urdan and Kaplan, 2020).

##### Protection mechanism against anxiety

A supplemental mechanism underlying decreasing value over time could be the regulation of negative exam-related emotions. If students, in line with the arguments pertaining to expectancy above, increasingly realize the difficulties they face while studying for the exam (i.e., experience declines in expectancy), they may increasingly experience negative emotions such as anxiety, frustration or shame (Turner and Schallert, 2001; Pekrun, 2006). Indeed, recent research has found that high situative expectancy positively predicted positive achievement-related emotions such as hope and negatively predicted negative emotions such as frustration (Berweger et al., 2022). This assumption is also compatible both with our findings regarding increasing performance avoidance motivation and previous findings that exam-related anxiety increases as exams approach (Pekrun et al., 2009; Rottweiler et al., 2018). Declining self-reported attainment value could thus indicate students’ explicit devaluation of the exam as an emotion regulation strategy of their increasing anxiety (for further discussion, see Davis et al., 2008, and Tamir et al., 2015). This regulation mechanism could potentially “mask” any psychological effects of temporal discounting, which should show an increasing subjective importance of the exam.

Overall, it seems promising to further investigate the contribution of different motivational qualities to study behavior – for example, by investigating the exact relationship of the value component and its sub-facets to (performance-) approach and avoidance motivation or by drawing on Self-Determination Theory and investigating to which degree externally regulated motivation exam contributes to study efforts for an exam (Ryan and Deci, 2000). In particular, it would be interesting to test this approach against the idea that students discount the value of an exam in order to regulate their anxiety.

#### Expectancy, value, and behavior energization

In addition to the mechanisms suggested above, it is conceivable that the role of motivational expectancy and value beliefs in energizing study behavior changes in other ways as well. Specifically, we discuss the potential roles of (i) relative motivation and of (ii) urgency or pull effects close to a deadline.

First, various authors (e.g., Atkinson, 1957; Hofer and Fries, 2016; Achtziger and Gollwitzer, 2018; Capelle et al., 2022) suggested that not “absolute” study motivation (as measured by most instruments) is the crucial factor determining study behavior but rather study motivation relative to other activities. The mechanism underlying the different trajectories of motivation and studying could then be that while absolute study motivation declines, it becomes stronger *relative to other action alternatives*. For example, students could primarily study to avoid experiencing should-conflicts with regard to studying: Other activities might simply become less attractive because they are increasingly “tainted” by students’ bad conscience for not studying (Capelle et al., 2022). Note that such an assumption is consistent with the idea that study behavior is mainly driven by performance avoidance motivation as well as the trajectories of both want- and should-conflicts we find (*cf.* Table 6).

Secondly, and related to the multiple action alternative perspective, Zhu et al. (2018) described a “mere urgency effect” in consumer choice research: Under spurious task urgency, people tend to work on tasks that are urgent, even if that means forgoing tasks with objectively better payoff. The authors explain this phenomenon with an attentional shift away from the value of a task outcome toward an impending deadline. Applied to the present context, this could mean that the value component is “decoupled” from energizing behavior close to the deadline. Indeed, in research on student motivation, Capelle et al. (2022) speculated that an upcoming deadline creates a situational pull which “takes over” the energization of study behavior from motivational beliefs: Like an object caught by the gravitational field of another object, individuals are thus almost passively “pulled towards” working on a task that is related to a deadline. While both concepts could explain a situative exception from temporal discounting (i.e., a decoupling of subjective task value and behavior), they are distinct in that the mere urgency effect is theoretically concerned with situations in which individuals may act against their priorities, while the situational pull is mainly concerned with a shift in the (subjective) source of behavior energization (which also sets it apart from Field Theory, e.g., Lewin, 1938). It thus seems interesting to consider the relationship of study motivation and study activities in terms of relative study motivation as well as the possibility of either a mere urgency effect or a situational pull.

#### Potential between-person moderators

We have so far discussed changes in motivation and study behavior as trajectories of the average student over time, which is reflected in our growth curve models. However, as we also found substantial variance in these trajectories as well as some significant predictors of between-person differences in how motivation and study behavior changes over time. It thus makes sense to briefly address potential between-person moderators in the future in order to increase the predictive power of growth models related to motivation and deadlines.

##### Motivational orientations

Relatively stable trait-like motivational orientations like competence beliefs, self-efficacy, or academic self-concept and general value orientations are likely to explain some of the between-person variance in motivation trajectories (Eccles et al., 1983; Wigfield and Eccles, 2000). Similarly, differences in trait-like performance approach and performance avoidance goal orientation as well as mastery-goal orientations are likely to explain some of the between-person variance, in particular regarding motivation quality (Heimpel et al., 2006; Robinson et al., 2008; Corr and Krupić, 2017).

##### Trait self-control

Another obvious candidate moderator is trait self-control, as it is related to impulsivity, i.e., the sensitivity to the temporal proximity of consequences (e.g., Steel and König, 2006) and with goal conflicts (Fujita, 2011; Inzlicht et al., 2021). Self-control could either facilitate studying in the absence of declining motivation or directly “protect” against the decline of motivation (Schmeichel et al., 2010; Milyavskaya et al., 2015). In particular, it would be interesting to see if students actually apply more self-control strategies as a deadline comes closer (Grund and Carstens, 2019). Moreover, it is conceivable that students who apply few or no self-control strategies rely on the onset of either a mere urgency effect or a situational pull to energize their studying.

##### Future time perspective

Another probable between-person moderator is future time perspective (e.g., Nuttin and Lens, 1985; Simons et al., 2004). Similar to the impulsivity aspect of self-control, future time perspective describes the individually differing inclination to foresee future consequences of one’s actions. Individual differences in future time perspective have been related to individually varying motivation to study (e.g., Lens et al., 2012). We would thus expect individuals with a long future time perspective to exhibit a flatter declines in study motivation compared to individuals with a shorter future time perspective as well as earlier onsets of study activities.

##### Pacing style

An additional likely between-person moderator primarily related to the trajectories of studying before a deadline is pacing style (Gevers et al., 2009). Pacing styles denote different patterns in how individuals allocate time during task completion. Recent research found different profiles of pacing styles with almost half of students showing a “deadline action pacing style” which is characterized as investing substantially more study effort toward a deadline (Konradt et al., 2021). However, evidence regarding the relationship between different pacing styles and academic outcomes is mixed (Vangsness and Young, 2020; Hartwig and Malain, 2022).

### Potential practical consequences: how to set and frame deadlines

Although our findings should be replicated and tested for causality before informing educational decisions, the present research is potentially relevant for educational practitioners. Deadlines are frequently used as a tool to motivate students to study (Ariely and Wertenbroch, 2002; Bjørnebekk and Gjesme, 2009). While our research supports the notion that students study more toward a deadline on average, it also suggests that deadlines might be a double edged sword. Specifically, deadlines may come at the cost of increasing performance avoidance motivation and potentially even anxiety as the exam or deadline comes closer (Burgess et al., 2004; Rottweiler et al., 2018). In the short term, this may mean increased levels of stress and loss of interest among students (Amabile et al., 1976). In the long term, this may accumulate into decreasing grades and university dropout. For example, Robinson et al. (2019) report that students whose expectancy and value beliefs declined more rapidly over time were more likely to drop out of an engineering major. However, considering the substantial individual differences between students, it seems likely that different groups of students are affected differently by deadlines (e.g., Ostermaier, 2018; Dietrich et al., 2019; Konradt et al., 2021). For example, deadlines might be motivationally helpful for students with (initially) little motivation to study but less so for those with high study motivation. Indeed, it even seems conceivable that even increased performance avoidance motivation or stress before an exam might be overall preferable to some students if the alternative means not being able to muster any motivation at all and therefore failing an exam, which in turn could jeopardize the chances to achieve a degree at all (Kosovich et al., 2017). One approach which could enable educators to reap the benefits of deadlines and also foster (or at least protect) more beneficial motivational qualities might be to frame deadlines in terms of an opportunity to gain and apply new knowledge and skills so as to foster beneficial forms of motivation (Jackson, 2002). A supplemental approach might be to substitute “high stakes” exams at the end of the semester with several low-stakes assessments throughout the semester (Cafarella, 2014; but see Theobald et al., 2021; Benden and Lauermann, 2022).

### Limitations and future research

The current research has several limitations. First, while more robust and ecologically valid than cross-sectional designs, our research design is correlative and thus warrants no causal inferences. Secondly, several single item measures were used in the study. Given the diversity of constructs assessed repeatedly over several weeks, this made data collection more feasible and likely increased participant retention (e.g., Wanous et al., 1997; Allen et al., 2022). At the same time, single item constructs cannot account for change due to measurement error, so true change in these constructs has to be assumed (e.g., Geiser, 2012). Moreover, we measured only parts of the overall construct sets of the relevant motivational theories. While this approach allowed us to generate fairly broadly applicable insights into the temporal dynamics of motivation, it may have had the cost of overlooking more nuanced changes. This possibility should be addressed in future research. For example, in the present research, value was assessed with one item that most closely resembles attainment value in expectancy-value theories (e.g., Eccles and Wigfield, 2020). Future research could assess temporal changes in all positively valenced subfacets of task value related to an exam in addition to negatively valenced costs. Indeed, despite their high correlations, recent research has identified some differential associations between value subfacets and differential outcomes (e.g., Perez et al., 2019; Robinson et al., 2019; Berweger et al., 2022). In particular the inclusion of the cost-component might uncover additional insights (Emanuel et al., 2022). Likewise, the consideration of mastery goals, possibly while distinguishing between mastery-approach and mastery-avoidance goals (e.g., Elliot and McGregor, 2001; Muis and Edwards, 2009; Jagacinski et al., 2010; Han, 2016), as well as students’ use of metacognitive learning strategies (e.g., Pintrich et al., 1993; Efklides and Vauras, 1999) might uncover additional insights into how students’ motivation changes as a temporal landmark draws closer. Thirdly, study time was assessed via weekly self-report measures and might thus have been subject to recollection bias or context effects. Still, due to the weekly measurement occasions, these biases are likely lower than in most other research settings, which are, for example, completely invisible in cross-sectional research designs (e.g., Trull and Ebner-Priemer, 2014). Moreover, as mentioned previously, our sampling period included the Christmas holidays at the end of the year, raising the question how readily our results transfer to other exam preparation periods. It is difficult to say how this might have affected our results. For example, it seems possible that students may have restored their psychological resources and thus gained study motivation during the holiday season which might have led us to underestimate declines in expectancy and value. Conversely, students might have felt more time pressure when they resumed their studies after the holidays which may have exaggerated the declines we observed. Given the consistency of our findings with previous results as well as the results of our nonlinear models (*cf.* Figures 5, 6), we are fairly confident that our results can be replicated in an assessment period without holidays. Finally, it is unclear to which degree our findings generalize to other student populations. On the one hand, our sample was fairly heterogenous as it comprised students from three different lectures in two different courses of study which implies a certain degree of generalizability of our overall findings. At the same time, our conditional growth curve models already identified some differences in the trajectories of the focal variables, mainly between courses of study and students of different age. Still, other lecture-specific characteristics which we did not explicitly consider might be associated with differential changes of motivation and study behavior before an exam. For example, in our sample, lecturers in the two education science courses were more experienced (*ca.* 15–20 years teaching experience) than the lecturer in the psychology statistics course (*ca.* 5 years teaching experience) which might in part be reflected in the differences between study courses we found. Other potentially relevant, but fairly elusive variables include exam difficulty and teaching style. Given that the lectures in our sample also shared some features (e.g., all had one high stake exam at the end of the semester) and our sample included only undergraduate students (i.e., Bachelor’s degree) with a majority of female students, it remains to be seen whether the temporal dynamics we discovered can also be found for other temporal landmarks (e.g., deadlines for a writing assignment) or other courses of study.

## Conclusion

The present research pursued two central objectives. First, to investigate if and how university students’ motivation and study behavior systematically change as a temporal landmark (i.e., an upcoming exam) comes closer. Second, to investigate an apparent discrepancy in the current literature on achievement motivation which has so far documented declining expectancy and value beliefs over time (e.g., Kosovich et al., 2017; Dietrich et al., 2019; Robinson et al., 2019; Benden and Lauermann, 2022) and the current behavioral economics literature which has so far found increasing study behavior over time among university students (e.g., König and Kleinmann, 2005; Liborius et al., 2019; Capelle et al., 2022). Both strands of research differ in several methodological aspects. In addition to different time periods covered and different levels in the specificity of measurement (e.g., task- or course-specific), temporal landmarks and temporal discounting effects are rarely considered in educational psychology research (*cf.* Trope and Liberman, 2003; Steel and König, 2006). We thus modeled the trajectories of both expectancy and attainment value beliefs and study behavior in parallel and under equal conditions over actual passing time using parallel growth curve models. Additionally, we considered changes in motivation quality based on motivation valence (i.e., performance approach and performance avoidance motivation) and study quality (i.e., use of deep and surface learning strategies) as well as changes in motivational conflict experience. Overall, our results indicate that even under the same conditions, expectancy and attainment value beliefs decline while students’ study time increases as an exam comes closer in time. Moreover, our results suggest that students’ studying is increasingly driven by performance avoidance motivation (i.e., fear of performing badly or failing) as the exam draws closer. This is consistent with students’ increasing use of surface learning strategies, although a switch from deep to surface learning strategies is likely also adaptive before exams. Likewise, students increasing study efforts are accompanied by a decline in the experience of should conflicts and an increase in the experience of want conflicts, suggesting that their study efforts come at the cost of other personal goals and needs. As the present research provides a first glimpse at the dynamics of students’ motivation and study behavior before a temporal landmark, these results should be considered as priors for future research investigating these dynamics in more detail. Fundamentally, we believe that the present research convincingly shows the relevance of temporal landmarks for systematic variations in students’ situative motivation and study behavior. Given that time is a rare predictor in psychology that is truly exogenous and objectively measurable, we would like to encourage others to consider the temporal distance of key events in future research on motivation and study behavior.

## Data availability statement

The raw data supporting the conclusions of this article will be made available by the authors, without undue reservation.

## Ethics statement

The studies involving humans were approved by Ethics Committee of Bielefeld University (EUB). The studies were conducted in accordance with the local legislation and institutional requirements. The participants provided their written informed consent to participate in this study.

## Author contributions

JC: conceptualization, data curation, writing – original draft, writing – review & editing, formal analysis, methodology, and visualization. KS: data curation, investigation, and writing – review and editing. SF: writing – review and editing and supervision. AG: conceptualization, data curation, funding acquisition, investigation, writing – review and editing, supervision, and methodology. All authors contributed to the article and approved the submitted version.

## Footnotes

^1^Note that in the present research, we use the term “performance approach motivation” synonymously with “pursuing a performance approach goal” and the term “performance avoidance motivation” synonymously with “pursuing a performance avoidance goal.”
